# Analysis of spontaneous labor progression of breech presentation at term

**DOI:** 10.1371/journal.pone.0262002

**Published:** 2022-03-14

**Authors:** Ines Benmessaoud, Margot Jamey, Barbara Monard, Jean-Patrick Metz, Aude Bourtembourg-Matras, Rajeev Ramanah, Didier Riethmuller, Abdellah Hedjoudje, Nicolas Mottet

**Affiliations:** 1 Department of Obstetrics and Gynecology, Jean Minjoz Hospital, Besançon University Medical Center–Alexander Fleming Boulevard, Besançon, France; 2 Department of Obstetrics and Gynecology, University Hospital Grenoble Alpes, Grenoble, France; 3 EA 3290 Group, University of Franche-Comte–Alexander Fleming Boulevard, Besançon, France; 4 Nanomedecine Laboratory, EA4662, University of Franche-Comte, Besançon, France; Lausanne University Hospital: Centre Hospitalier Universitaire Vaudois (CH), SWITZERLAND

## Abstract

**Background:**

Cervical dilatation curves are widely used to describe normal and abnormal labor progression for cephalic presentation. Labor curves for breech presentations have never been described.

**Objectives:**

The aims of this study were to examine the pattern of labor progression in women with a breech presentation and to determine whether the type of breech or parity can influence the speed of cervical dilatation.

**Study design:**

We analyzed the labor data from 349 women with a term, singleton, and breech fetus after spontaneous onset of labor in 2010–2018. Cesarean deliveries were excluded. The patterns of labor progression were described by examining the relationship between the elapsed times from the full dilatation and cervical dilatation stages. Average labor curves were developed using repeated-measures analysis with 3^rd^ degree polynomial modeling. The results were interpreted according to parity and the type of breech.

**Results:**

The first stage of labor progression was divided into a latency phase from 0 to 5 cm of dilatation and an active phase from 5 to 10 cm. In the active phase, the median speed of cervical dilatation was 1.67 cm/h [1.25, 2.61] (2 cm/h for multipara and 1.54 cm/h for nullipara). The difference by parity was significant in the active phase (p< 0.05). The cervical dilatation rate from 3 cm to 10 cm did not significantly differ between the complete and frank breeches (1.56 cm/h vs 1.75 cm/h, p = 0.48). However, the median cervical dilatation rate from 8 cm to complete dilatation was faster for complete breeches (1.92 cm/h versus 1.33 cm/h, p = 0.045).

**Conclusion:**

As with cephalic presentation, the first stage of labor progression for breech presentation can be divided into a latent and active phase. Labor progression should be interpreted with respect to parity, and women should be informed that the type of breech does not seem to influence the cervical dilatation rate when there is adequate management.

## Introduction

Cervical dilatation curves are widely used to describe normal and abnormal labor progression. Referential data from the Consortium on Safe Labor for spontaneous deliveries are used for the management of labor progression to optimize recourses for obstetric interventions, such as the use of oxytocin and cesarean section [1, 2]. These data serve as a reference to define normal values for the first stage of labor progression, which can be divided into a latent phase and an active phase, which does not start until at least 5–6 cm of dilatation is observed [1]. Friedman was the first to report mean labor curves by dividing the labor process into several stages and phases [3, 4]. Because of the methodological limitations of these preliminary studies, Zhang et al. reported contemporary curves developed with repeated-measures regression and polynomial function. In this retrospective study, the 95^th^ percentile rate of active phase dilatation for a vertex presentation varied from 0.5 cm/h to 0.7 cm/h for nulliparous women and from 0.5 cm/h to 1.3 cm/h for multiparous women.

However, these previous studies excluded nonvertex presentation, and no labor curves have been established for breech presentation at term. Because there is insufficient data in the literature, it is not possible to recommend specific labor durations for breech delivery. Moreover, it is difficult to conclude whether recommendations concerning oxytocin use for labor arrest described for cephalic presentation are also applicable for breech labor management [5]. Contemporary data about cervical dilatation in this specific situation may be useful for reducing cesarean deliveries for these pregnancies.

The first objective of this study was to describe the progression of labor with breech presentation and the spontaneous onset of labor. The secondary objectives were to determine whether parity and type of breech (frank or complete) can influence the cervical dilatation speed.

## Materials and methods

We conducted a single-center retrospective cohort study of consecutive term breech deliveries from 1 January 2010 to 31 December 2018 at a single academic teaching hospital. Under French regulations, the study was exempt from institutional ethics review because it was a retrospective observational study using anonymized data from medical records. The women were systematically informed that the obstetrical and neonatal data could be used for the evaluation of medical practices and were explicitly informed that they could sign an opposition form.

Women were eligible after 37 weeks of gestation with a live breech presentation, spontaneous onset of labor and a vaginal delivery. We excluded cesarean deliveries and home births. During the study period, an attempt of vaginal breech delivery at term was attempted if the following hospital-specific guidelines were met: normal findings for low-dose CT pelvimetry in nulliparous women (anteroposterior inlet diameter ≥ 105 mm, transverse diameter ≥ 115 mm, bispinous diameter ≥ 95 mm and a Magnin Index ≥ 23), a clinical adequate maternal pelvis for multiparous women, no fetal head deflexion, and no suspected fetal macrosomia.

Concerning labor management, epidural analgesia was recommended, and oxytocin augmentation without labor induction was allowed to induce regular uterine contractions, if needed. The latency phase was a period characterized by painful uterine contractions and variable changes in the cervix with some degree of effacement and slow progress of dilatation up to 5 cm. The active phase of labor was a period characterized by regular painful uterine contractions, a significant degree of cervical effacement and dilatation up to 5 cm. The management protocol is available in the S1 Appendix.

Detailed demographic data were extracted from the patients’ records, including their medical and obstetrical history (maternal age, parity, body mass index). We collected detailed labor and delivery information from the medical files and electronic partograms (ePartogram, DIAMM® software, version 8.7 Rev 14), including the cervical examination time, cervical dilatation (3 to 10 cm) and station at each examination, total duration of the first and second stages, duration of the expulsive efforts, use of oxytocin, type of anaesthesia, type of breech presentation (frank breech or incomplete breech and complete breech), birth injury perineum, episiotomy and total volume of bleeding. We included incomplete breech in the frank breech group. The following neonatal data were collected: the umbilical arterial pH, arterial lactates, the Apgar score at 5 minutes (min), the birthweight, the head circumference and whether the newborn was transferred to the intensive care unit.

We characterized the labor progression patterns by examining the relationship between the elapsed times from the full dilatation and cervical dilatation stages, as previously described by Zhang et al. [6, 7]. Because the participants were admitted at various cervical dilatation stages, we performed interval-censored regression analyses, with 10 cm of dilatation as the starting point, moving backward in time. We constructed average labor curves using repeated-measures analysis with 3^rd^ degree polynomial modeling. A 3^rd^ order polynomial had the best fit for our cervical dilatation data. We characterized the labor durations (min) by examining the distribution of time intervals from one cervical dilatation stage to the next and ultimately to full dilatation. The median, first quartile and third quartile of the cervical dilatation velocity were calculated.

The patients were divided into two groups according to parity: nulliparous and multiparous (> 1). For each group, the average duration of cervical dilatation has been collected. Thus, the median cervical dilatation rate to the full dilatation (cm/h) has been calculated. The cervical dilatation curves of the first stage of labor were created. The cervical dilatation curves were also described with respect to the type of breech presentation. The qualitative variables were compared using the chi-squared test. The normality of the quantitative variables was tested by the Kolmogorov-Smirnov test. In accordance with the results of this test, the statistical significance of the differences in the qualitative variables was tested using the paired Student t-test or paired Mann–Whitney U test. Pearson’s correlation coefficient (r2) between the mean values was calculated. Analyses were performed by R software (online at http://www.R-project.org, the R Foundation for Statistical Computing, Vienna, Austria). For the continuous data, the variables are presented as the mean value ± standard deviation (SD) or the median and interquartile range. The categorical variables are presented as frequencies and percentages. All reported p values are two-tailed. P values of less than 0.05 were regarded as statistically significant.

## Results

**Fig 1:**
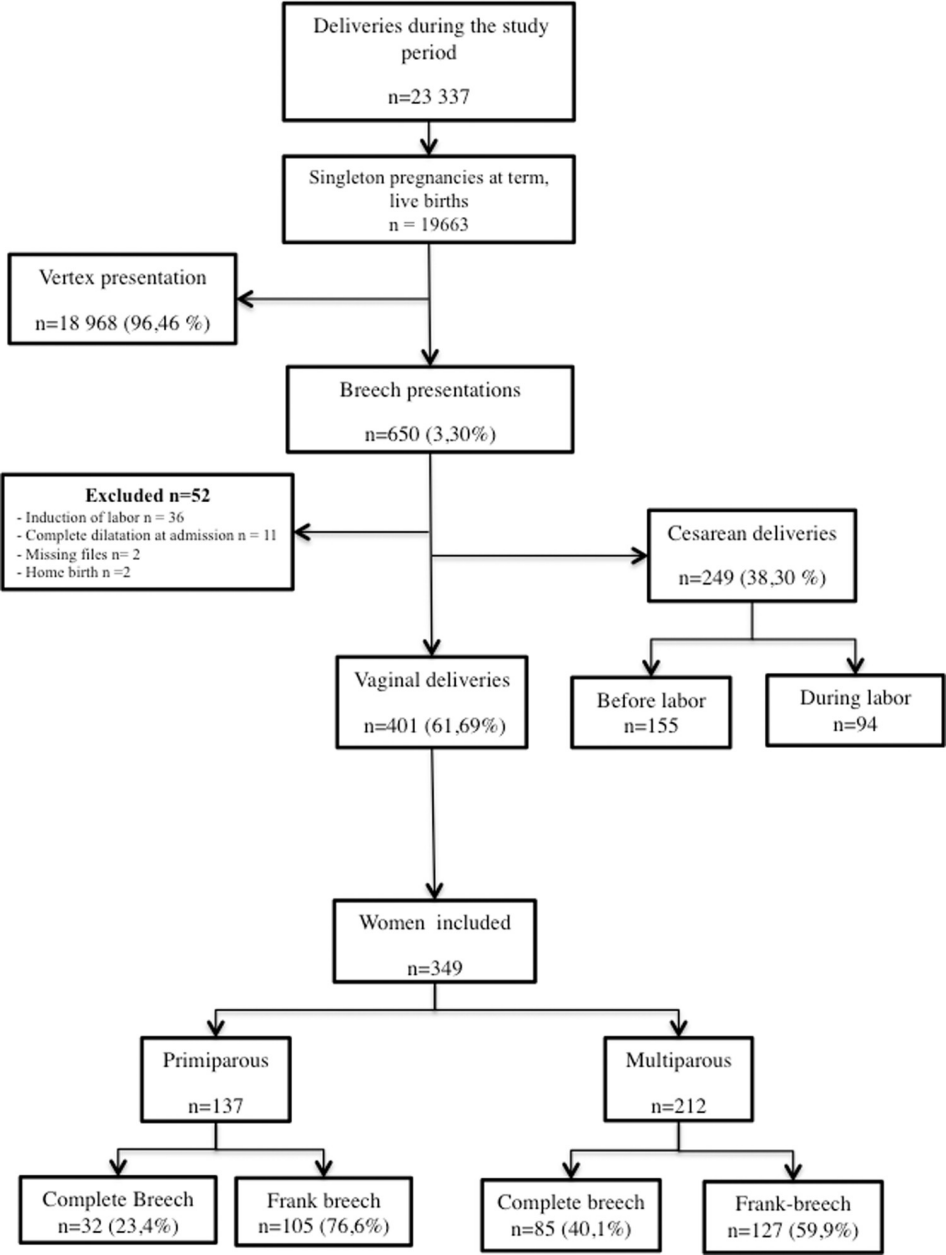
Flow chart.

Out of the 23,337 women who delivered at the hospital during the study period, 18 968 (96,46%) had cephalic presentations, and 650 (3,3%) had breech presentations. Two hundred forty-nine (38.3%) women had a cesarean breech delivery: 155 before labor and 94 during labor. Among the 495 attempts of vaginal breech delivery, 401 ultimately resulted in a vaginal delivery. Fifty-two breech deliveries were excluded. Finally, 349 patients were included in the study: 137 were primiparous, and 212 were multiparous. The most common type of breech presentation was complete in both the multiparous (n = 127) and nulliparous groups (n = 105) (Fig 1). The maternal and neonatal characteristics are summarized in the Table 1. Frank breech presentations were significantly more common in the primiparous patients (p = 0.002) (Table 1). Oxytocin was introduced in the latent phase for 39 patients (14.4%), in the active phase for 150 patients (55.5%) and finally during the 2nd stage for 81 patients (30%).

**Table 1:** Baseline characteristics of the study population by parity.

	Multiparous (n = 212)	Nulliparous (n = 137)	p-test
Maternal age at delivery (years)	**31.32 (4.93)**	**28.57 (4.15)**	**<0.001**
Gestational age at delivery (weeks)	39.52 (1.16)	39.38 (1.10)	0.264
Prepregnant BMI (kg/m^2^)	22.96 (5.22)	22.28 (4.29)	0.201
Epidural analgesia (n, (%))	197 (92.9)	134 (97.8)	0.019
**Oxytocin use (n, (%))**	**154 (72.6)**	**116 (84.7)**	**0.013**
Type of breech presentation: (n, (%))			
• Complete Breech • Frank and incomplete breech	85 (40.1)	32 (23.4)	0.002
	127 (59.9)	105 (76.6)	
Postpartum hemorrhage: (n, (%))			0.837
• hemorrhage > 500 ml and < 1L • hemorrhage > 1L	8 (3.8)	7 (5.1)	
	2 (0.9)	1 (0.7)	
Birth injury perineum: (n, (%))			0.671
Absence of any lesion	45(52.9)	55 (57.3)	
First degree	23 (27.1)	24 (25.0)	
Second degree	12 (14.1)	14 (14.6)	
Third degree	4 (4.7)	2 (2.1)	
Fourth degree	1 (1.2)	0	
**Episiotomy (n, (%))**	**5 (2.4)**	**18 (13.1)**	**<0.001**
Newborn birth weight (grams)	3135.28 (428.34)	3024.05 (402.24)	0.016
Newborn Head circumference (cm)	33.81 (4.92)	34.09 (3.23)	0.561
Newborn Apgar score at 5 minutes (mean (SD))	9.78 (0.70)	9.73 (0.77)	0.505
**Umbilical arterial pH (mean (SD))**	**7.16 (0.11)**	**7.13 (0.13)**	**0.013**
**Arterial lactates (mean (SD))**	**5.22 (2.37)**	**6.54 (2.60)**	**<0.001**
Newborn transfer: (n, (%))			0.563
• Neonatal unit • Neonatal intensive care unit	4 (10.0)	3 (8.1)	
	3 (7.5)	1 (2.7)	

**Fig 2:**
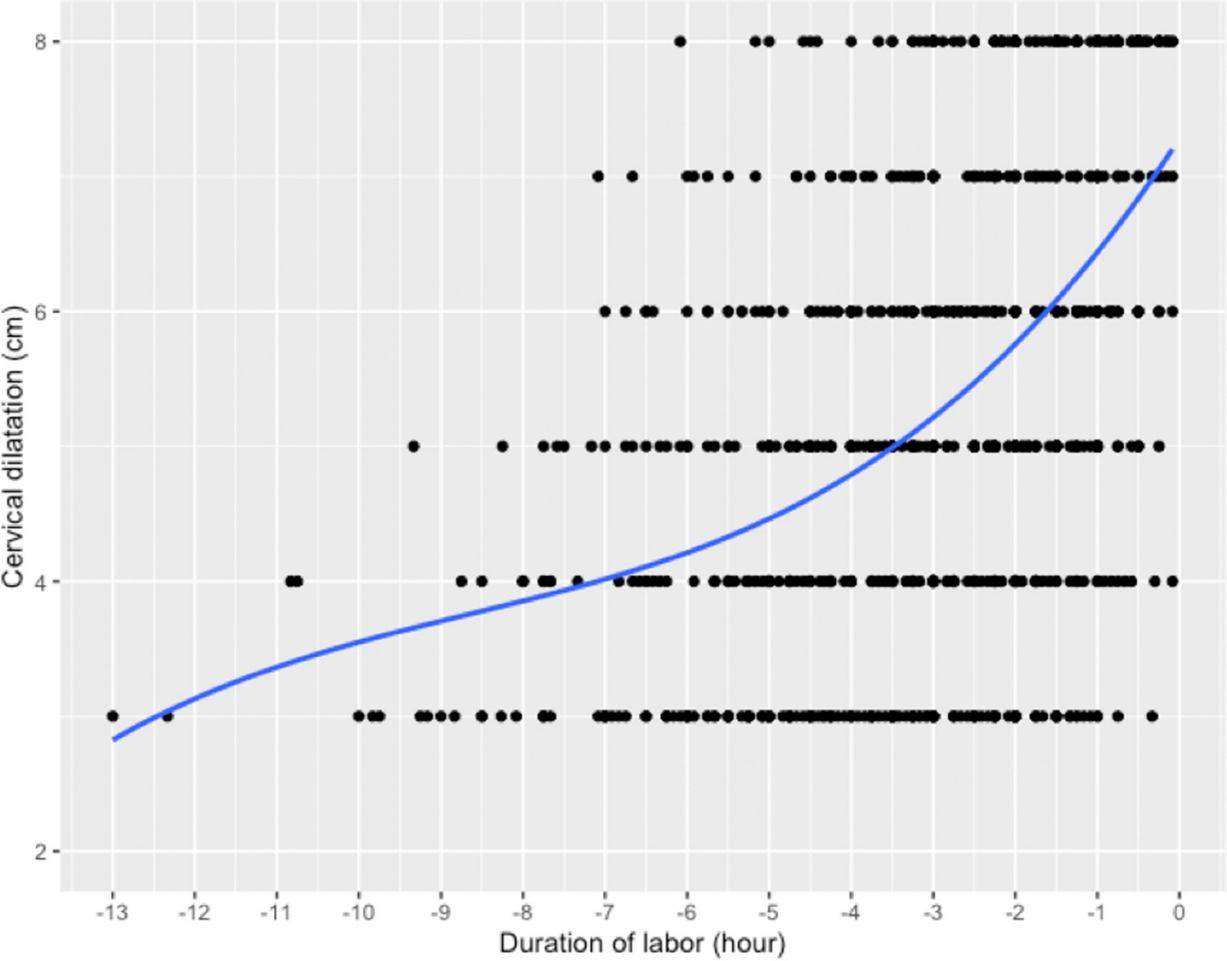
Cervical dilatation curves of breech presentation with spontaneous labor and vaginal deliveries.

In the whole cohort, the first stage of labor could be divided into a latency phase from 0 to 5 cm of dilatation and an active phase from 5 to 10 cm of dilatation. The maximal slope in the rate of change in cervical dilatation over time did not start until at least 5 cm was observed (Fig 2). The cervical dilatation rate was slower than 1 cm/hour in the latency phase and faster than 1 cm/hour in the active phase (Table 2). The median velocity of cervical dilatation from 3 to 10 cm was 1.65 cm/h [1.27, 2.59]. In the active phase, from 5 to 10 cm, the median speed of cervical dilatation was 1.67 cm/h [1.25, 2.615] (Table 3).

**Table 2:** Number of patients (n) analyzed and the median dilatation rate from one cervical dilatation stage to the next for primiparous and multiparous females. Results are expressed as median (IQR).

Cervical Dilatation stage (cm)	Multiparous (n = 212)	Nulliparous (n = 137)	Median dilatation rate p-test	Number of patients p-test
3 to 4	0.83 [0.67, 1.00], n = 38	0.80 [0.65, 1.00], n = 35	0.337	0.337
4 to 5	0.86 [0.70, 1.00], n = 42	1.00 [0.81, 1.00], n = 34	0.125	0.125
5 to 6	1.00 [1.00, 1.00], n = 40	1.00 [0.67, 1.00], n = 32	0.074	0.074
6 to 7	1.00 [0.92, 1.00], n = 37	1.00 [0.75, 1.00], n = 25	0.222	0.222
7 to 8	1.00 [0.83, 1.00], n = 39	1.00 [0.80, 1.00], n = 21	0.703	0.703
8 to 9	1.00 [0.80, 1.00], n = 49	1.00 [0.86, 1.00], n = 33	0.956	0.956
9 to 10	1.00 [0.92, 2.00], n = 99	1.00 [0.86, 1.33], n = 63	0.167	0.167

**Table 3:** Median cervical dilatation rate to the full dilatation (cm/h) for overall and according to type of breech presentation and parity. Results are expressed as median (IQR).

Cervical dilatation (cm)	Overall (n = 349)	Complete breech (n = 117)	Frank breech (n = 123)	*p*	Multiparous (n = 212)	Nulliparous (n = 137)	*p*
3 to 10	1.65 [1.27, 2.59] n = 179	1.56 [1.21, 2.57], n = 52	1.75 [1.29, 2.63], n = 127	0.482	1.73 [1.33, 2.73] n = 100	1.62 [1.17, 2.33] n = 79	0.256
4 to 10	1.80 [1.20, 2.67 n = 147	1.71 [1.20, 2.40], n = 50	2.00 [1.26, 2.67], n = 97	0.257	2.00 [1.27, 3.00] n = 87	1.48 [1.13, 2.22] n = 60	0.053
5 to 10	1.67 [1.25, 2.61] n = 183	2.00 [1.25, 3.10], n = 67	1.67 [1.18, 2.50], n = 116	0.316	**2.00 [1.31, 3.08] n = 110**	**1.54 [1.11, 2.22] n = 73**	**0.045**
6 to 10	1.60 [1.11, 2.78] n = 142	1.50 [1.23, 3.39], n = 54	1.75 [1.00, 2.38], n = 88	0.386	1.70 [1.13, 3.43] n = 80	1.50 [1.10, 2.00] n = 62	0.210
7 to 10	1.50 [1.00, 2.94] n = 138	1.50 [1.15, 2.40], n = 44	1.50 [1.00, 3.00], n = 94	0.716	1.50 [1.00, 2.40] n = 82	1.50 [1.00, 3.00] n = 56	0.622
**8 to 10**	1.39 [0.98, 2.67] n = 160	**1.92 [1.00, 3.57], n = 52**	**1.33 [0.91, 2.22], n = 108**	**0.045**	1.60 [1.00, 2.67] n = 99	1.33 [0.92, 2.03] n = 61	0.445
**9 to 10**	[0.86, 1.93] n = 162	**1.05 [1.00, 2.96], n = 52**	**1.00 [0.86, 1.33], n = 110**	**0.017**	1.00 [0.92, 2.00] n = 99	[0.86, 1.33] n = 63	0.167

**Fig 3:**
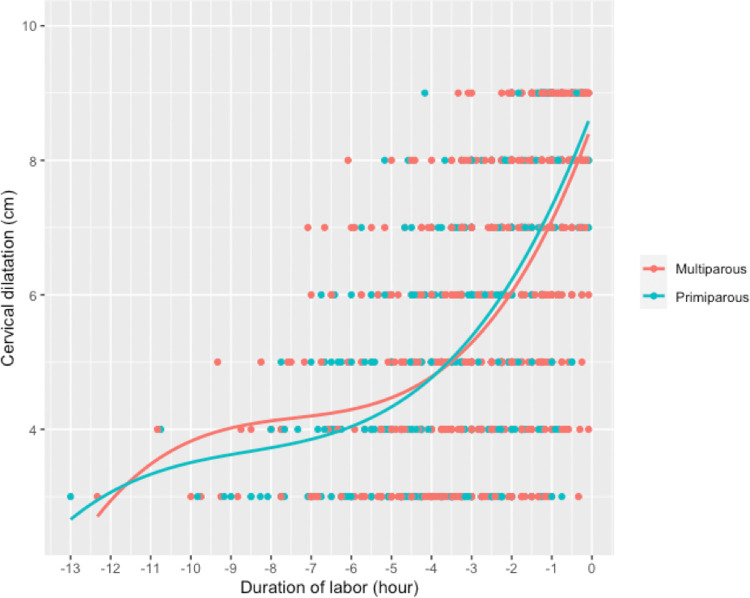
Cervical dilatation curves for the primiparous and multiparous women.

The percentage of patients admitted at early stages of dilatation was higher in the primiparous women: 79 (57.7%) primiparas and 100 (47.2%) multiparas were admitted from 3 cm of cervical dilatation (Table 3). For each one-centimeter interval of cervical dilatation, there was no significant difference in the number of patients assessed between the two groups (Table 2). Cervical dilatation was faster in the multiparous women than in the primiparous women and accelerated as labor progressed in both groups (Fig 3). From 5 to 10 cm, the median cervical dilatation time was 202 min for the primiparas and 178 min for the multiparas (p = 0.12) (Table 4). The median speed of cervical dilatation in the active phase (5 to 10 cm) was 1.54 cm/h [1.11, 2.22] for the primiparas and 2.00 cm/h [1.31, 3.08] for the multiparas (p <0.05) (Table 3).

**Table 4:** Time (min) required from one cervical dilatation stage to the full dilatation. Results are expressed as mean and standard deviation.

Cervical dilatation (cm)	Multiparous (Min)	Nulliparous (Min)	p-test
3 to 10	251,46 (129,30)	276,14 (141,28)	0,225
4 to 10	211,36 (126,25)	246,17 (122,86)	0,099
5 to 10	178,10 (105,97)	202,33 (100,13)	0,123
6 to 10	153,89 (97,24)	170,31 (89,01)	0,302
7 to 10	137,98 (89,55)	126,61 (76,67)	0,439
8 to 10	94,79 (69,47)	97,36 (61,83)	0,813
9 to 10	57,89 (38,72)	61,75 (34,15)	0,519

**Fig 4:**
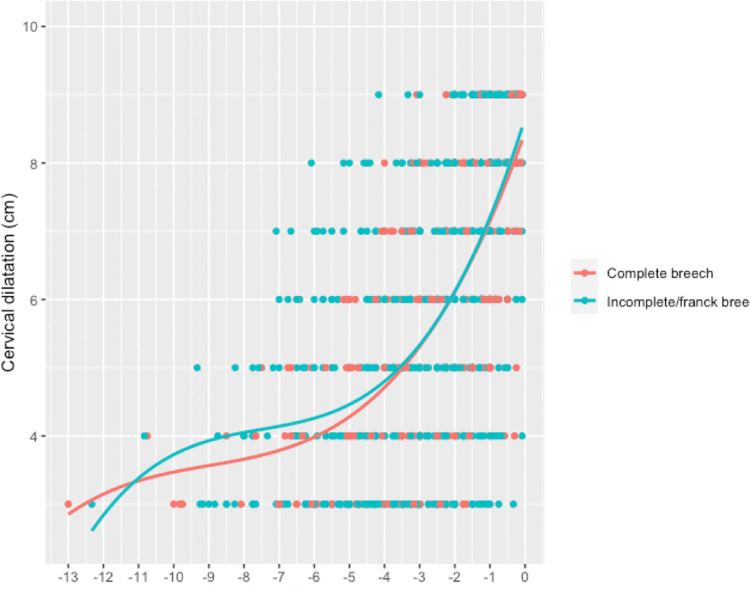
Cervical dilatation curves according to the type of breech presentation.

The rate of cervical dilatation from 3 cm to 10 cm was not significantly different between the complete and frank breech groups (1.56 cm/h vs 1.75 cm/h, p = 0.48) (Table 3). However, when the labor curves were juxtaposed in the active phase, and median cervical dilatation from 8 cm to complete dilatation was observed to be faster for complete breeches (1.92 cm/h versus 1.33 cm/h, p = 0.045) (Table 3, Fig 4).

## Discussion

### Principal findings

This study is the first to propose labor curves of breech presentation at term in spontaneous labor and highlights that the first stage of labor can also be divided into a latent and active phase. In the cohort in our study, the latent phase lasted until cervical dilatation reached 5 cm and gradually accelerated from 5 to 10 cm during the active phase. Cervical dilatation was faster at the beginning of the latency phase in the multiparas, and the start of the active phase was progressive for both groups. According to parity, we found that both the latency phase and the active phase were faster for the multiparous women. The difference by parity was significant in the active phase (p< 0.05) but no significant differences were found in the rate of cervical dilatation across the types of breech.

### Clinical implications

The American College of Obstetricians and Gynecologists (ACOG) and the American Society of Maternal Fetal Medicine (SMFM) have proposed guidelines to manage cervical dilatation during labor for cephalic presentations. The active phase is defined to begin from 6 cm, and neither prolonged nor stopped labor should be diagnosed before 6 cm of dilatation. Cesareans should be performed in the absence of progression for 4 hours after the administration of oxytocin if the uterine activity is satisfactory and the membranes are ruptured or in the absence of progression for 6 hours after the administration of oxytocin if uterine activity is not satisfactory [2]. The absence of a consensual definition of labor progression during breech presentation and the lack of standard protocols can lead to unnecessary cesarean sections being performed. In 2006, only 18/30 maternities included in the PREMODA study were able to provide a protocol in which conditions for an attempt at breech vaginal delivery were detailed. The rate of vaginal deliveries ranged from 1.7 to 49.7%, depending on the center, and there was no threshold to define an abnormally long first stage of labor [8].

### Results

In 1997, a study involving 266 patients with a breech presentation reported an average labor duration of 460 min [9]. In our study, the average cervical dilatation time from 5 to 10 cm was 202 min for the primiparas and 178 min (p = 0.1) for the multiparous, which were consistent with the results of the PREMODA study since the duration of the active phase was less than four hours for 66.2% of the women included. The use of oxytocin augmentation during spontaneous labor can explain this difference. Indeed, the rate of oxytocin augmentation was approximately 77% in our study and was similar to that of the PREMODA study (70%) (8). This high rate could reflect a lack of tolerance by the medical team toward a prolonged latent phase for breech delivery compared to cephalic presentation.

It is important to distinguish the total duration of labor and cervical dilatation speed during both the latent and active phases. According to the British guidelines for breech delivery, the first stage of labor should be managed with the same principles as with a cephalic presentation [10]. Oxytocin may be considered for a contraction frequency fewer than four in ten, and a cesarean section should be offered when the progress of labor is slow, regardless of parity. However, this decision should take into account that the cervical dilatation speed seems to spontaneously differ in breech delivery cases by the stage of first labor and parity.

It is interesting to compare the median duration of labor for cephalic presentation reported in the National Collaborative Perinatal Project and our data for breech presentation [11]. The median cervical dilatation time from 5 to 10 cm was 2.1 (h) for nulliparous patients with a cephalic presentation and was 3.36 h in our study. In the Zhang et al. study, the median velocity of cervical dilatation for the nulliparous patients was 1.2 cm/h at 5 cm and 2.8 cm/h at 8 cm [6]. Thus, the cervical dilatation rate in nulliparous women seems to be slower for breech presentations than for cephalic presentations, despite the high rate of oxytocin use in our study (77%). In our study oxytocin was introduced in the latent phase for 39 patients (14.4%), in the active phase for 150 patients (55.5%) and finally during the 2nd stage for 81 patients (30%). The use of oxytocin is described in the protocol. Only 14.4% of patients receive oxytocin in latent phase. But in view of the new recommendations and these first studies describing breech labor, a reflection on the use of oxytocin can be considered (5).

In our study, 3.15% of newborns were transferred, of which 2.00% (n = 7) were transferred to neonatology and 1.15% (n = 4) to intensive care unit. These rates are lower than reported in the French national perinatal survey in 2016 (4.2% in neonatology and 2,4% in intensive care units) (12). The arterial pH was significantly lower with an average of 7.13 in nulliparous versus 7.16 in multiparous (p = 0.013). Concerning the 4-newborn transferred to the intensive care unit, 3 had an arterial pH < 7 and one had an arterial pH of 7.20 but the Apgar score was > 7 at 5 min.

The cervical dilatation rate in the multiparous women remained faster than that in the nulliparous women throughout the entire labor period. These data should be taken into account before abnormal first-stage labor progression is diagnosed for breech presentation after the spontaneous onset of labor. Recently, a comparative study between frank and complete/incomplete breeches provided evidence that perinatal morbidity was not associated with the fetal leg posture in intended vaginal deliveries. There was no significant difference in the rate of cesarean section indicated for labor arrest between the frank and complete breech groups or in the duration of the first stage of labor [12]. The median velocity of cervical dilatation from 3 cm to 10 cm did not differ between the complete and frank breeches. Although complete breech presentation can be regarded as unfavorable for vaginal delivery, our results suggest that complete breech presentation can also be a good dilator pole for the cervix when pelvic accommodation is optimal. In the active phase, cervical dilatation was faster from 8 cm to full dilatation for complete breeches.

### Research implications

In current practice, some exclusion criteria for a vaginal birth approach may be opinion based, and cesarean section is often offered for cases with complete breech because of a high risk of cord prolapse or poor cervical dilatation. When women expecting a breech baby undergo counseling, it is important to inform them that a frank or a complete breech does not significantly influence labor progression during the first stage of labor.

### Limitations

There are some limitations that should be considered when interpreting our results. In this study, women who underwent a cesarean delivery were excluded such as the Zhang’s study, which can lead to selection bias (1). According to labor progression analyses reported in the current literature, cervical dilatation was slower in the studies that included patients who had a cesarean section. Since women undergoing cesarean section in the first stage are more likely to have a prolonged labor period, their exclusion likely shortened the total duration and duration for progression in one centimeter increments [2]. Moreover, some practices have changed over the ten years of this study, particularly concerning the administration of oxytocin during labor. In our study, the use of oxytocin probably had an impact on labor duration since the median duration was 4.6 h in the nulliparous women and was 6.9 h in the study by Zhang about the contemporary patterns of spontaneous labor [1]. Oxytocin for augmentation was used in nearly half of the women included in the Consortium on Safe Labor and 74.9% in the PREMODA Study (8). It is possible that the high rate of labor augmentation reported in our study suggests a more prophylactic than a therapeutic role. A reflection on the high-level use of oxytocin should be considered and discussed in specific recommendations about management of breech delivery.

### Conclusions

Our results confirm that the first stage of labor for breech presentation can be divided into two phases: the latent phase, from 0 to 5 cm, and the active phase, from 5 to 10 cm. During attempts of vaginal breech delivery, labor progression should be assessed according the latent and the active phases, and according the parity. Women should be informed that the type of breech does not seem to significantly influence the cervical dilatation during spontaneous labor.

## Supporting information

S1 Appendix Protocol for attempted vaginal breech delivery. (DOCX)

S1 File Partograph. (PDF)

S2 File Characteristics. (PDF)
