# Supplementary material for: Analysis of spontaneous labor progression of breech presentation at term
Source: PLoS One. 2022 Mar 14;17(3):e0262002. doi: 10.1371/journal.pone.0262002 (PMC8920216; doi:10.1371/journal.pone.0262002)
Supplement: S1 Appendix — (DOCX) [file pone.0262002.s001.docx]

**S1 Appendix. Protocol for attempted vaginal breech delivery.**

Criteria for accepting a vaginal delivery attempt of breech presentation:

- Presentation of the medical records at the obstetrical staff
- No vaginal delivery post-term pregnancies in case of nulliparous
- Weight below 90th percentile
- Pelvimetry around 37 weeks' gestation after failure, contraindication or refusal of external cephalic version (ECV)
- Pelvimetry analyze

- analysis of the basin morphology

- analysis of measures

- Pelvimetry for multiparous women with a history of childbirth is not systematic and is often discussed in obstetrical staff

Labor management:

^1st^ stage of labor: Cervical dilation (passive and active phase)

- Fetal heart rate analysis upon admission by midwife and obstetrician
- Obstetrician is systematically informed of the medical records for breech presentation
- Hourly monitoring of cervical dilation by the midwife with a vaginal examination
- Hourly annotation of labor monitoring parameters on a partograph :

- Cervical dilation, rate of contractions, type of presentation, height of presentation, information of the amniotic sac, colors of the amniotic fluid, patient's pain, analgesia used, administered treatment, use of syntocinon, foetal heart rate (FHR), constant medical,

- Epidural is recommended
- Artificial rupture of the amniotic sac in the absence of labor progress

(no change in the dilation in cm in 1 hour of labor)

- Late artificial rupture of the amniotic sac if complete breech presentation
- Administration of oxytocin

- If no evolution of the cervix over 1 hour, measurement in cm

- If insufficient rate of uterine contractions (expected rhythm 3-4 UC/10 min)

- Stop of oxytocin administration

- If FHR anomaly

- If hyperkinesia during labor

2nd stage of labor: The engagement phase and expulsive efforts

- Administration of oxytocin

- Systematic administration of oxytocin in the second phase of labor (descent and expulsion phase)

- Oxytocin at the beginning of expulsive efforts to limit them

- Beginning of expulsive efforts when the presentation is as low as possible

Stared expulsive after at least 2 or 3 complete hours if the FHR is correct

The

- Presence of the obstetrician at the expulsive efforts
- Maneuvering delivery in the systematic presence of the obstetrician

Type of obstetric maneuvers : Lovset or Suzor’s maneuvers , Mauriceau's maneuver, Bracht’s maneuver, Demelin's maneuver

- Presence of available instruments in the delivery room such as spatulas or forceps in case of head retention

Formation : Trained Team

- Regular training of the medical team in maneuvers on mannequins
- Regular training in the interpretation of pelvimetry in staff
- Conducting breech birth studies and analysis of the center’s data
