# Supplementary material for: Analysis of spontaneous labor progression of breech presentation at term
Source: PLoS One. 2022 Mar 14;17(3):e0262002. doi: 10.1371/journal.pone.0262002 (PMC8920216; doi:10.1371/journal.pone.0262002)
Supplement: S1 File — (PDF) [file pone.0262002.s002.pdf]

3 cm	4 cm	5 cm	6 cm	7 cm	8 cm	9 cm
7:15 PM		9:00 PM			10:00 PM	
				12:15 AM	1:15 AM	
	3:15 AM			3:45 AM		
8:00 AM				9:00 AM	10:30 AM	
	5:00 PM			6:00 PM		
	5:00 PM		7:00 PM			8:00 PM
4:00 AM	4:45 AM		6:00 AM	7:00 AM	8:45 AM	9:00 AM
				1:00 PM		
6:45 AM		8:15 AM		9:15 AM	9:45 AM	10:15 AM
4:00 AM		5:00 AM	6:00 AM			7:00 AM
		10:00 AM				
10:15 PM	10:30 PM	11:30 PM		12:15 AM		12:45 AM
	2:00 AM		3:00 AM		4:00 AM	4:45 AM
			12:10 PM	1:15 PM	2:30 PM	3:30 PM
					4:00 AM	
	10:30 PM					11:45 PM
3:20 AM	4:45 AM	5:05 AM			6:10 AM	
12:15 AM						1:15 AM
11:20 PM		12:45 AM	1:45 AM	4:45 AM	5:15 AM	
		4:00 PM		5:00 PM		
						1:00 AM
6:20 AM				7:20 AM		10:15 AM
	3:00 AM			3:45 AM		
				6:00 AM	6:30 AM	
		2:30 AM			3:30 AM	4:30 AM
12:30 AM	1:15 AM		2:15 AM		3:15 AM	
11:30 PM		12:30 AM				1:15 AM
	3:00 AM			4:00 AM		5:00 AM
8:30 PM					9:30 PM	
	10:15 AM	11:15 AM				
				6:15 AM		6:45 AM
9:45 AM					11:00 AM	11:45 AM
12:30 AM						1:45 AM
7:00 PM		8:00 PM	9:00 PM		10:00 PM	
11:30 AM			12:30 PM			1:30 PM
			4:15 PM		5:15 PM	
		6:30 AM		7:30 AM		
9:00 AM	10:15 AM	11:15 AM	12:15 PM		1:15 PM	
11:00 AM		12:00 PM			1:00 PM	
8:45 PM	9:45 PM	10:45 PM			11:00 PM	
					12:45 AM	
					5:15 PM	

	8:00 PM		8:30 PM		9:00 PM	
	7:45 PM				8:15 PM	8:45 PM
	6:15 PM		7:15 PM			8:15 PM
		8:00 AM				
	10:45 PM					11:15 PM
11:00 PM	12:30 AM	1:00 AM		1:15 AM		
				8:30 AM	9:30 AM	10:30 AM
3:45 AM	4:45 AM	6:30 AM		7:30 AM		
2:30 AM						
6:15 AM	8:15 AM		10:00 AM			
9:30 AM		11:00 AM				
10:00 PM					11:00 PM	12:00 AM
3:00 PM	4:45 PM		5:45 PM		6:45 PM	8:00 PM
	7:00 PM		8:00 PM			
1:30 AM		2:45 AM				
6:30 AM		8:00 AM				
9:00 AM		9:45 AM				
		1:45 PM			2:45 PM	
12:00 AM	1:00 AM				3:00 AM	3:30 AM
			1:30 AM			2:15 AM
8:30 AM	9:30 AM	10:30 AM			11:30 AM	
12:30 PM			1:30 PM	2:30 PM		
12:00 PM		1:00 PM	2:00 PM	3:00 PM	4:00 PM	
	1:15 AM			2:15 AM	3:15 AM	
		4:30 AM	5:30 AM			6:00 AM
11:15 PM			12:30 AM			
	8:30 PM			9:15 PM		
	11:15 PM			12:15 AM		1:15 AM
4:15 AM		5:45 AM	6:45 AM		7:50 AM	9:00 AM
10:30 AM		11:45 AM				
					4:30 AM	
1:15 AM			2:05 AM		3:15 AM	
3:30 AM	4:30 AM	5:30 AM		6:30 AM		7:45 AM
1:30 PM	2:15 PM	3:45 PM	5:00 PM	6:00 PM	8:00 PM	9:00 PM
						3:30 AM
	8:15 PM	9:00 PM				
9:15 AM	10:15 AM	11:30 AM				12:45 PM
		9:30 PM		10:30 PM		
					8:15 PM	
10:05 PM	10:25 PM					10:45 PM
	6:45 AM		7:50 AM		8:45 AM	10:45 AM
1:00 PM	2:00 PM			3:00 PM		

			12:45 AM			12:50 AM
1:45 AM	2:45 AM					
	9:30 PM			10:30 PM		
	2:30 PM	3:45 PM	5:00 PM	6:45 PM		
1:00 AM	2:00 AM	3:00 AM				
4:00 AM				5:00 AM	6:00 AM	7:00 AM
	9:00 PM				9:10 PM	
4:00 AM	5:15 AM	6:15 AM	6:45 AM	7:45 AM	8:45 AM	
	3:30 PM	4:30 PM	5:45 PM		6:45 PM	7:45 PM
		2:00 AM				3:30 AM
		6:00 PM	7:00 PM	8:00 PM		
	4:40 PM	6:00 PM		7:15 PM		8:15 PM
	9:00 AM				10:00 AM	
12:00 PM				1:30 PM		
2:30 AM	3:30 AM		4:30 AM			
		12:45 PM			1:30 PM	
10:15 PM	11:30 PM	12:30 AM	12:40 AM	1:10 AM		
7:00 AM						8:00 AM
			5:00 AM		6:00 AM	
	12:30 PM	1:30 PM	2:00 PM	3:00 PM	4:45 PM	
11:00 PM		12:00 AM				
	9:00 PM	10:00 PM	11:00 PM	12:00 AM		1:00 AM
	4:05 AM	6:00 AM	7:45 AM	8:45 AM	9:45 AM	10:45 AM
4:00 PM					4:45 PM	
		4:00 AM				
			7:15 PM	8:15 PM		9:15 PM
		1:30 AM				
	8:00 AM		9:00 AM			10:00 AM
2:45 AM		4:00 AM				
6:00 PM	7:00 PM	8:15 PM		9:15 PM		
	11:15 PM					
	4:00 AM	4:40 AM			5:15 AM	
	13h50					
					7:00 PM	
3:00 AM		4:00 AM			5:00 AM	
	1:30 PM	2:30 PM		3:30 PM		
	8:15 PM			9:40 PM		
6:00 PM	7:00 PM	8:00 PM	9:00 PM			
5:30 AM						
4:30 PM			5:30 PM	6:30 PM	8:00 PM	9:00 PM
		3:15 PM	4:15 PM		4:35 PM	
	10:00 AM		11:00 AM	11:30 AM		12:30 PM
		8:00 AM	9:30 AM			

		7:00 AM	8:00 AM		9:00 AM	10:00 AM
10:15 PM					11:45 PM	
		6:00 AM		7:00 AM		
9:00 PM			10:15 PM			11:09 PM
11:00 AM		12:30 PM			1:30 PM	
8:00 AM	9:30 AM		10:40 AM			
5:00 PM	6:00 PM	7:00 PM				
11:00 AM		12:00 PM	1:15 PM	2:00 PM	3:00 PM	3:45 PM
12:00 PM			1:15 PM			2:30 PM
6:00 PM		7:45 PM		9:00 PM		10:20 PM
	3:15 AM		4:45 AM			6:00 AM
			11:30 AM			12:30 PM
		2:00 PM	3:00 PM			3:45 PM
	1:30 PM		2:45 PM			
3:30 AM				5:00 AM		
11:00 AM	11:40 AM		12:45 PM		1:15 PM	1:30 PM
		3:30 AM		4:30 AM		
		11:00 AM				12:15 PM
			8:00 PM		9:30 PM	10:45 PM
5:30 AM	7:00 AM	8:00 AM	9:00 AM			10:00 AM
	4:30 AM					5:45 AM
					4:15 PM	
1:00 PM		2:00 PM			3:00 PM	5:00 PM
		4:00 AM	5:00 AM		6:00 AM	7:00 AM
3:00 AM	5:00 AM		6:00 AM	7:00 AM		
6:30 AM	8:00 AM	9:15 AM	10:15 AM			11:10 AM
						10:15 AM
10:45 PM						
		1:15 PM	2:15 PM		3:20 PM	4:15 PM
	3:30 AM		4:30 AM		5:30 AM	
		5:15 PM			5:45 PM	
	4:00 AM		5:45 AM	6:45 AM		8:00 AM
9:30 PM				11:00 PM		
		10:30 AM			11:30 AM	
					7:15 AM	
		4:45 AM				
4:00 AM	5:20 AM	6:30 AM	7:30 AM	8:30 AM	9:30 AM	
3:00 AM	4:00 AM	6:30 AM	7:00 AM	8:00 AM		8:20 AM
8:30 AM		10:00 AM	11:00 AM	12:00 PM	1:00 PM	
3:00 PM	4:30 PM	5:30 PM		6:30 PM		
9:30 AM		11:00 AM			12:15 PM	1:15 PM
4:00 AM	5:00 AM					5:45 AM
	2:00 AM		3:00 AM	4:00 AM		

8:30 PM	9:15 PM		10:30 PM	11:15 PM		
8:15 PM						9:30 PM
3:45 AM	5:15 AM	7:15 AM			7:45 AM	
	6:30 AM			7:30 AM	8:30 AM	
10:00 AM	10:45 AM	11:50 AM		2:00 PM		3:00 PM
9:30 PM		10:45 PM	11:45 PM	12:30 AM	1:30 AM	
3:30 PM		4:15 PM	4:30 PM		4:45 PM	5:05 PM
			10:15 PM			
2:45 AM		3:45 AM		5:00 AM		
		9:30 AM		11:00 AM	12:00 PM	1:00 PM
				5:00 AM		
					4:00 AM	5:30 AM
2:30 AM					3:30 AM	
10:45 PM	11:30 PM	12:45 AM	1:50 AM			
2:20 PM		4:00 PM	5:00 PM	6:10 PM	7:15 PM	
8:00 PM	9:05 PM			11:05 PM		
2:15 AM						
					12:40 PM	
5:30 AM						6:15 AM
	9:15 AM	10:45 AM		11:45 AM	12:45 PM	1:45 PM
1:15 AM			2:15 AM			2:45 AM
	5:00 PM		6:00 PM	7:00 PM		8:15 PM
	2:30 AM	3:15 AM				
		12:15 PM	1:15 PM			
7:00 AM	8:15 AM	9:15 AM	10:15 AM	11:15 AM	12:20 PM	12:40 PM
8:45 PM					11:15 PM	
9:15 PM						10:15 PM
		2:30 PM		4:00 PM		5:00 PM
		11:00 AM	12:00 PM	1:00 PM	2:00 PM	3:00 PM
8:15 PM		9:30 PM	10:30 PM			
9:00 PM	10:10 PM	11:15 PM	12:15 AM		1:15 AM	
10:30 AM			10:45 AM			
1:45 PM	3:15 PM	4:30 PM			5:35 PM	6:35 PM
11:15 PM	12:15 AM		1:30 AM			
						12:30 AM
		2:15 AM		3:15 AM	4:15 AM	
	3:30 PM	4:45 PM		5:45 PM	6:45 PM	7:45 PM
9:00 PM						
6:30 AM	7:30 AM	8:00 AM			8:50 AM	
12:15 AM		2:15 AM	3:15 AM	5:15 AM	7:45 AM	
				8:30 AM	9:30 AM	11:30 AM
			1:45 PM			
	2:30 AM			3:15 AM		

	9:45 PM		11:45 PM	1:15 AM	2:15 AM	3:30 AM
	6:30 AM	8:00 AM		9:00 AM	10:15 AM	
2:45 PM	4:00 PM	6:10 PM	8:10 PM	9:45 PM		
	12:45 PM					
4:00 AM		5:30 AM		6:45 AM		
2:45 AM		4:00 AM				
	2:30 PM	4:45 PM	6:00 PM	7:00 PM		8:00 PM
5:30 AM			6:30 AM		7:30 AM	8:30 AM
6:45 PM			7:45 PM			8:45 PM
9:00 PM		10:15 PM		11:15 PM		12:30 AM
8:30 PM		9:45 PM		10:45 PM		
9:45 AM	10:50 AM	12:00 PM	1:00 PM	2:00 PM		3:00 PM
9:45 PM						10:45 PM
12:00 AM						
4:45 AM				5:45 AM		
2:15 AM			3:30 AM	4:30 AM		7:00 AM
		5:35 AM		6:15 AM	7:15 AM	8:30 AM
6:15 AM						
			11:50 PM		12:16 AM	
7:45 AM						
		12:00 AM			1:30 AM	2:30 AM
9:30 AM		11:30 AM	1:30 PM			2:40 PM
	2:55 PM		4:05 PM	5:00 PM	6:15 PM	
		12:30 PM				2:15 PM
	11:30 PM		12:50 AM			
		11:45 PM				
	6:15 AM		7:00 AM		8:05 AM	9:05 AM
		11:50 PM	12:20 AM		12:50 AM	
2:30 AM	4:50 AM	6:05 AM		7:05 AM		
10:30 AM			11:30 AM		12:30 PM	1:30 PM
12:30 AM		2:00 AM	3:00 AM	4:00 AM	5:00 AM	5:25 AM
		7:10 AM	8:15 AM	9:10 AM		9:19 AM
7:20 AM	8:30 AM	10:30 AM	11:40 AM	12:40 PM		2:00 PM
12:45 AM	2:18 AM	3:15 AM			5:20 AM	6:30 AM
	3:00 PM	4:00 PM		6:00 PM		
		1:00 PM				
	4:00 AM			4:30 AM		4:50 AM
7:50 PM	9:30 PM		10:30 PM	11:30 PM		
8:40 AM	10:15 AM	11:15 AM		12:15 PM	1:15 PM	2:45 PM
		10:10 PM			11:10 PM	
	9:15 PM			10:15 PM	11:15 PM	12:15 AM
11:35 PM		12:35 AM		1:45 AM	2:45 AM	5:30 AM
4:15 PM	5:15 PM	7:15 PM			8:15 PM	

				5:15 AM		
		5:00 PM		6:15 PM	7:15 PM	7:45 PM
12:30 AM		1:45 AM			2:50 AM	3:50 AM
7:50 AM		9:00 AM	10:00 AM	11:00 AM	12:00 PM	1:00 PM
11:00 AM	1:20 PM	2:20 PM	3:49 PM		4:35 PM	5:30 PM
11:05 AM	11:30 AM	12:30 PM	1:30 PM	3:40 PM	4:40 PM	5:50 PM
		9:40 AM		11:00 AM		
		11:15 AM		12:30 PM		
4:00 AM		5:30 AM	6:30 AM	8:00 AM	9:00 AM	10:00 AM
			1:30 AM		2:45 AM	3:45 AM
9:50 PM	10:50 PM		11:50 PM		12:50 AM	1:50 AM
6:15 AM	7:45 AM	8:45 AM	9:45 AM		10:45 AM	11:45 AM
2:05 AM		3:15 AM			5:30 AM	6:10 AM
		5:45 AM	6:45 AM		7:10 AM	7:22 AM
9:00 AM		10:15 AM	11:15 AM	12:15 PM		1:15 PM
	5:15 AM	5:40 AM				
10:30 AM		11:30 AM			12:30 PM	
					4:11 AM	
4:00 PM		5:00 PM	5:40 PM	6:45 PM	8:00 PM	10:20 PM
2:30 AM	4:20 AM	5:30 AM			9:45 AM	
	9:20 PM		10:50 PM	11:50 PM	12:50 AM	1:50 AM
		2:45 PM			3:30 PM	3:37 PM
5:45 PM			6:50 PM			7:50 PM
10:30 PM			11:40 PM		12:50 AM	
2:00 PM	3:30 PM	4:30 PM	5:30 PM		10:00 PM	
	6:30 PM		7:15 PM		8:15 PM	9:15 PM
7:00 AM	11:00 AM	12:00 PM			1:15 PM	2:24 PM
11:15 AM			12:30 PM	1:35 PM	2:45 PM	4:30 PM
	10:40 PM				12:05 AM	
		6:30 AM			8:00 AM	10:15 AM
10:40 PM		11:45 PM		1:00 AM	2:00 AM	
	9:45 AM	11:15 AM	12:15 PM			1:15 PM
3:30 PM				5:15 PM		6:25 PM
2:30 AM						3:50 AM
11:15 AM		12:50 PM	2:00 PM			3:00 PM
	10:00 PM		11:15 PM	11:40 PM	12:00 AM	2:45 AM
7:00 AM	9:15 AM	11:10 AM	12:10 PM	1:15 PM	2:15 PM	3:15 PM
	5:30 AM	6:30 AM	8:00 AM	9:20 AM	12:20 PM	
			6:10 AM	7:25 AM		8:30 AM
		7:45 AM	9:20 AM	11:45 AM		12:45 PM
11:00 AM	1:15 PM		8:00 PM		9:00 PM	10:00 PM
	8:15 PM	9:30 PM		10:30 PM	2:25 AM	
	2:25 AM				3:45 AM	

9:15 AM		11:00 AM			12:00 PM	
	8:00 PM					9:30 PM
9:15 AM		11:00 AM				
					5:15 PM	
6:15 AM		8:00 AM	10:00 AM			
5:45 AM					8:00 AM	9:00 AM
		4:45 AM		5:20 AM	5:55 AM	
3:30 PM					5:00 PM	
	8:10 PM	9:20 PM		10:15 PM	12:30 PM	2:55 AM
3:15 AM	5:15 AM	7:15 AM			8:15 AM	9:15 AM
3:00 AM	4:30 AM	5:30 AM	6:30 AM			
1:15 AM		2:30 AM	3:30 AM	4:30 AM	5:40 AM	9:15 AM
		10:00 PM				11:15 PM
7:50 AM				8:55 AM	11:00 AM	12:00 PM
		5:30 PM		7:15 PM	8:15 PM	9:15 PM
		1:00 AM				
11:55 AM			1:15 PM	2:15 PM	3:15 PM	4:15 PM
		2:30 AM				3:45 AM
	10:40 PM		11:40 PM			
	9:30 PM	10:15 PM	2:20 AM			
	5:20 PM	6:28 PM	9:00 PM	10:00 PM		
		1:10 AM				
				12:00 PM		
		9:00 AM		10:15 AM	11:40 AM	3:00 PM
7:45 AM		9:15 AM			10:45 AM	
		9:40 AM		10:40 AM		
2:45 AM			4:00 AM			
						6:45 AM
						3:40 PM
8:00 AM			10:10 AM	11:30 AM		2:00 PM
8:00 AM	9:20 AM	10:30 AM		11:45 AM		12:40 PM
2:30 PM	4:15 PM	5:30 PM	8:00 PM	9:00 PM		10:00 PM
	5:30 AM		7:00 AM	8:00 AM	10:00 AM	12:30 PM
2:15 AM	3:25 AM				4:30 AM	
1:45 AM	3:45 AM	4:45 AM		7:45 AM		
	1:00 PM				2:40 PM	3:50 PM
		9:15 PM			10:25 PM	11:25 PM
10:50 AM	12:00 PM	1:00 PM	2:00 PM	3:20 PM	4:20 PM	6:10 PM
	2:00 PM		4:30 PM			
10:35 PM		12:00 AM	1:00 AM	2:00 AM		3:00 AM
			3:15 AM			
	12:30 PM	1:30 PM	2:30 PM		3:30 PM	4:30 PM
7:20 AM	10:30 AM	11:30 AM	2:30 PM	3:30 PM		

5:10 AM	6:40 AM	8:10 AM	10:30 AM	11:30 AM	12:30 PM	2:30 PM
5:00 AM	6:15 AM					
10:50 PM	12:45 AM	2:00 AM			3:15 AM	
9:30 PM	10:40 PM	11:40 PM				
3:50 AM		5:00 AM			6:00 AM	7:00 AM
6:15 PM		8:45 PM				9:45 PM

10 cm	Oxytocin	Type of breech
11:30 PM	1	franck
2:00 AM	1	franck
4:15 AM	0	complete
12:30 PM	1	franck
6:45 PM	1	franck
8:30 PM	1	franck
9:30 AM	1	complete
1:45 PM	1	franck
11:00 AM	1	franck
8:00 AM	1	franck
11:00 AM	0	complete
1:00 AM	1	complete
5:30 AM	1	franck
4:30 PM	1	franck
5:30 AM	1	franck
12:15 AM	1	complete
7:00 AM	1	franck
2:15 AM	0	franck
6:15 AM	1	franck
6:15 PM	1	franck
3:00 AM	1	complete
10:40 AM	1	franck
4:15 AM	1	franck
7:15 AM	1	franck
5:00 AM	1	franck
4:15 AM	1	franck
1:30 AM	0	franck
6:00 AM	1	franck
10:30 PM	1	franck
12:30 PM	1	franck
8:45 AM	0	complete
12:00 PM	0	complete
3:15 AM	1	franck
11:00 PM	1	franck
2:10 PM	0	complete
6:45 PM	1	franck
10:30 AM	1	franck
2:15 PM	1	franck
2:15 PM	1	franck
12:15 AM	1	franck
1:15 AM	0	complete
5:45 PM	0	complete

9:15 PM	1	franck
9:00 PM	0	franck
9:15 PM	1	franck
9:30 AM	1	franck
11:20 PM	0	complete
1:30 AM	1	franck
11:30 AM	1	franck
8:30 AM	1	franck
3:30 AM	0	complete
11:15 AM	1	franck
12:00 PM	1	franck
12:30 AM	1	complete
9:00 PM	1	franck
9:00 PM	1	franck
3:45 AM	1	franck
8:15 AM	1	complete
10:20 AM	1	franck
3:15 PM	1	complete
3:45 AM	1	franck
3:00 AM	1	franck
12:30 PM	1	franck
3:45 PM	0	franck
4:45 PM	1	complete
4:15 AM	1	complete
6:30 AM	0	complete
5:00 PM	1	complete
12:45 AM	1	franck
9:20 PM	1	franck
2:00 AM	1	franck
9:30 AM	1	complete
1:05 PM	0	franck
4:45 AM	1	franck
4:45 AM	1	franck
8:45 AM	1	franck
10:00 PM	1	franck
2:15 PM	1	franck
10:00 PM	1	complete
13h45	1	complete
11:30 PM	1	complete
8:30 PM	0	franck
11:10 PM	0	franck
12:00 PM	1	franck
3:20 PM	1	franck

1:15 AM	0	complete
3:45 AM	1	complete
11:30 PM	1	complete
7:15 PM	1	franck
4:00 AM	1	franck
7:15 AM	1	franck
9:18 PM	0	complete
9:45 AM	1	complete
9:00 PM	1	complete
4:30 AM	1	franck
9:00 PM	1	franck
9:15 PM	1	complete
11:00 AM	1	complete
3:30 PM	1	franck
5:30 AM	1	franck
2:15 PM	1	franck
1:40 AM	1	complete
9:00 AM	1	franck
6:15 AM	1	franck
5:15 PM	1	complete
12:45 AM	0	complete
2:00 AM	1	franck
11:45 AM	1	franck
5:15 PM	1	franck
5:30 AM	1	complete
10:00 PM	1	complete
2:30 AM	1	complete
11:00 AM	1	franck
5:45 AM	1	franck
9:30 PM	1	franck
12:45 AM	1	franck
5:45 AM	1	franck
2:30 PM	0	franck
7:15 PM	1	franck
5:30 AM	1	franck
5:00 PM	1	franck
10:40 PM	1	franck
9:50 PM	1	complete
6:15 AM	0	complete
10:30 PM	1	franck
4:45 PM	1	complete
12:45 PM	1	franck
10:30 AM	1	complete

11:15 AM	1	complete
12:20 AM	1	franck
8:00 AM	0	franck
12:30 AM	1	franck
3:00 PM	1	complete
11:10 AM	1	franck
9:15 PM	1	franck
5:00 PM	1	complete
3:00 PM	1	complete
11:30 PM	1	franck
7:00 AM	0	franck
12:45 PM	1	franck
5:00 PM	1	franck
3:45 PM	1	franck
5:45 AM	0	franck
2:00 PM	1	franck
5:30 AM	1	franck
1:15 PM	1	franck
11:45 PM	1	franck
11:00 AM	1	franck
6:30 AM	1	franck
5:15 PM	0	complete
6:00 PM	1	franck
9:00 AM	1	franck
8:00 AM	1	franck
11:20 AM	1	franck
11:00 AM	1	complete
12:15 AM	0	franck
7:20 PM	1	franck
6:30 AM	1	complete
6:45 PM	0	complete
9:00 AM	1	franck
11:10 PM	0	complete
11:45 AM	1	complete
8:45 AM	1	franck
5:15 AM	1	franck
10:00 AM	1	franck
9:30 AM	1	complete
2:00 PM	0	complete
7:45 PM	0	franck
2:45 PM	1	franck
6:00 AM	0	franck
5:00 AM	1	franck

12:15 AM	1	franck
9:45 PM	0	complete
8:30 AM	1	complete
9:05 AM	0	franck
3:30 PM	1	franck
2:45 AM	1	complete
5:15 PM	0	complete
10:30 PM	0	franck
6:00 AM	0	franck
2:00 PM	1	franck
5:30 AM	0	complete
6:30 AM	1	franck
4:45 AM	1	franck
2:20 AM	1	franck
8:15 PM	1	complete
12:45 PM	1	franck
3:45 AM	1	franck
12:45 PM	0	complete
7:30 AM	1	franck
2:45 PM	1	franck
3:30 AM	1	complete
9:15 PM	1	franck
4:15 AM	0	complete
1:45 PM	1	franck
12:45 PM	1	franck
12:40 AM	1	franck
10:30 PM	0	franck
6:05 PM	1	franck
4:00 PM	1	franck
11:45 PM	1	complete
1:45 AM	1	franck
10:50 AM	0	franck
7:45 PM	1	franck
3:00 AM	1	franck
1:15 AM	0	franck
4:20 AM	1	franck
9:45 PM	1	franck
10:30 PM	0	franck
9:45 AM	1	franck
8:45 AM	1	franck
12:30 PM	1	complete
2:45 PM	0	franck
4:00 AM	1	franck

5:30 AM	1	franck
11:30 AM	1	franck
10:50 PM	1	complete
2:30 PM	1	franck
8:30 AM	1	franck
5:00 AM	1	franck
9:00 PM	1	complete
8:58 AM	1	franck
9:45 PM	1	franck
1:30 AM	0	franck
12:58 AM	1	complete
4:00 PM	0	franck
11:45 PM	1	complete
1:10 AM	0	franck
6:45 AM	1	franck
9:00 AM	1	franck
9:30 AM	1	franck
7:15 AM	1	complete
12:40 AM	1	franck
9:45 AM	0	franck
3:30 AM	1	complete
3:45 PM	1	franck
8:00 PM	1	complete
3:30 PM	1	franck
2:00 AM	0	complete
12:45 AM	0	franck
9:50 AM	1	complete
1:35 AM	1	complete
8:00 AM	1	complete
3:30 PM	1	complete
5:35 AM	1	complete
9:28 AM	1	franck
3:00 PM	1	complete
7:35 AM	1	complete
9:25 PM	1	franck
1:45 PM	0	franck
5:10 AM	1	franck
12:30 AM	1	franck
3:45 PM	1	franck
1:00 AM	1	complete
1:15 AM	1	franck
8:50 AM	1	complete
9:15 PM	1	franck

6:20 AM	1	franck
8:45 PM	1	franck
4:50 AM	1	franck
2:00 PM	1	complete
6:45 PM	1	franck
6:50 PM	1	franck
12:00 PM	1	franck
1:30 PM	1	franck
11:00 AM	1	franck
7:55 AM	1	franck
2:55 AM	1	franck
12:45 PM	1	franck
6:29 AM	1	complete
7:37 AM	1	complete
2:40 PM	1	franck
6:10 AM	1	complete
1:30 PM	1	franck
4:19 AM	0	franck
11:00 PM	1	complete
10:15 AM	1	franck
3:00 AM	1	franck
4:03 PM	1	complete
8:50 PM	1	franck
1:50 AM	0	complete
12:00 AM	1	franck
10:45 PM	0	franck
3:16 PM	1	franck
6:15 PM	1	franck
12:56 AM	1	franck
11:10 AM	1	complete
4:45 AM	1	franck
3:00 PM	0	franck
6:30 PM	0	franck
5:00 AM	0	complete
4:20 PM	0	franck
2:53 AM	0	complete
3:50 PM	1	complete
1:10 PM	1	franck
9:30 AM	1	complete
1:45 PM	1	franck
12:00 AM	1	franck
2:35 AM	1	franck
4:00 AM	0	complete

1:30 PM	0	franck
10:30 PM	0	complete
1:20 PM	1	franck
5:38 PM	0	franck
10:50 AM	1	complete
10:00 AM	1	complete
7:00 AM	0	complete
6:15 PM	1	franck
4:55 AM	1	complete
10:15 AM	1	complete
7:45 AM	1	franck
10:15 AM	1	franck
12:00 AM	0	franck
2:05 PM	1	franck
10:15 PM	0	complete
2:30 AM	0	complete
5:15 PM	0	franck
5:00 AM	0	complete
1:25 AM	1	franck
3:10 AM	1	complete
11:00 PM	1	franck
3:00 AM	0	franck
1:30 PM	1	complete
4:10 PM	1	franck
11:30 AM	0	franck
12:00 PM	1	complete
5:10 AM	0	complete
6:55 AM	0	franck
3:50 PM	0	complete
3:00 PM	1	complete
1:00 PM	1	franck
12:15 AM	1	complete
1:30 PM	1	franck
5:20 AM	0	franck
6:45 AM	1	franck
5:20 PM	1	franck
12:25 AM	0	franck
8:00 PM	1	franck
5:30 PM	1	complete
3:50 AM	1	franck
4:40 AM	1	franck
4:53 PM	0	franck
5:10 PM	1	complete

5:30 PM	1	franck
6:20 AM	0	franck
4:30 AM	1	complete
12:40 AM	0	complete
8:00 AM	1	franck
10:45 PM	1	franck

Number	3 cm	4 cm	5 cm	6 cm	7 cm	8 cm
51059728	19:15		21:00			22:00
5526774959					0:15	1:15
5602530242		3:15			3:45	
5502172123	8:00				9:00	10:30
5600384320		17:00			18:00	
5513172125		17:00		19:00		
51241267	4:00	4:45		6:00	7:00	8:45
5539375461					13:00	
5525222757	6:45		8:15		9:15	9:45
5615971210	4:00		5:00	6:00		
5603588855			10:00			
5501703388	22:15	22:30	23:30		0:15	
5613684939		2:00		3:00		4:00
5514541037				12:10	13:15	14:30
5539110834						4:00
5619252638		22:30				
5613670286	3:20	4:45	5:05			6:10
5512014286	0:15					
5619196862	23:20		0:45	1:45	4:45	5:15
5605749935			16:00		17:00	
5531017495						
51156036	6:20				7:20	
5509872307		3:00			3:45	
5619855755					6:00	6:30
5519391138			2:30			3:30
5508946258	0:30	1:15		2:15		3:15
5515573277	23:30		0:30			
5502348541		3:00			4:00	
51193719	20:30					21:30
51161655		10:15	11:15			
5522842419					6:15	
5605069117	9:45					11:00
5620292154	0:30					
5609192223	19:00		20:00	21:00		22:00
51192154	11:30			12:30		
51144036				16:15		17:15
5525888219			6:30		7:30	
5620660855	9:00	10:15	11:15	12:15		13:15
51028829	11:00		12:00			13:00
51033467	20:45	21:45	22:45			23:00
5524747962						0:45
5515521343						17:15

5608371965		20:00		20:30		21:00
5532291128		19:45				20:15
5600591858		18:15		19:15		
5614632812			8:00			
5527571975		22:45				
5622182745	23:00	0:30	1:00		1:15	
5623896918					8:30	9:30
5605687388	3:45	4:45	6:30		7:30	
5624685143	2:30					
5623483656	6:15	8:15		10:00		
5611869928	9:30		11:00			
5624483665	22:00					23:00
51144011	15:00	16:45		17:45		18:45
5526069283		19:00		20:00		
5624728387	1:30		2:45			
5529264526	6:30		8:00			
5527923296	9:00		9:45			
5616317477			13:45			14:45
5517734357	0:00	1:00				3:00
5626267152				1:30		
5626749021	8:30	9:30	10:30			11:30
5503932166	12:30			13:30	14:30	
5615187530	12:00		13:00	14:00	15:00	16:00
5613684939		1:15			2:15	3:15
5600642681			4:30	5:30		
5612452130						
5505554894	23:15			0:30		
5628217559		20:30			21:15	
5508075379		23:15			0:15	
51136202	4:15		5:45	6:45		7:50
5609659641	10:30		11:45			
5628197553						4:30
5519714470	1:15			2:05		3:15
5541790155	3:30	4:30	5:30		6:30	
51127331	13:30	14:15	15:45	17:00	18:00	20:00
51031031						
51140607		20:15	21:00			
5627468336	9:15	10:15	11:30			
5520154509			21:30		22:30	
5627459747						20:15
5624767288	22:05	22:25				
5541850476		6:45		7:50		8:45
51083731	13:00	14:00			15:00	

5532954162				0:45		
51071598	1:45	2:45				
5619416730		21:30			22:30	
5619385711		14:30	15:45	17:00	18:45	
5522661250	1:00	2:00	3:00			
5507292208	4:00				5:00	6:00
5522881825		21:00				21:10
51054689	4:00	5:15	6:15	6:45	7:45	8:45
5538150837		15:30	16:30	17:45		18:45
5512349039			2:00			
5508716791			18:00	19:00	20:00	
5506456691		16:40	18:00		19:15	
5543191706		9:00				10:00
5507823583	12:00				13:30	
5622150009	2:30	3:30		4:30		
5519964145			12:45			13:30
5542296777	22:15	23:30	0:30	0:40	1:10	
5542542816	7:00					
5517724960				5:00		6:00
5529963532		12:30	13:30	14:00	15:00	16:45
5538395559	23:00		0:00			
5535436150		21:00	22:00	23:00	0:00	
51219078		4:05	6:00	7:45	8:45	9:45
5543867874	16:00					16:45
5607292942			4:00			
5602817101				19:15	20:15	
51216958			1:30			
5612064635		8:00		9:00		
51196473	2:45		4:00			
5619871216	18:00	19:00	20:15		21:15	
5512041467		23:15				
5501449370		4:00	4:40			5:15
5545693393		13h50				
51059728						19:00
5540091645	3:00		4:00			5:00
5546720482		13:30	14:30		15:30	
5513460394		20:15			21:40	
5619137450	18:00	19:00	20:00	21:00		
5546596507	5:30					
5544693788	16:30			17:30	18:30	20:00
5546650562			15:15	16:15		16:35
7770		10:00		11:00	11:30	
5545093512			8:00	9:30		

51181836			7:00	8:00		9:00
5542021036	22:15					23:45
5516963310			6:00		7:00	
13256888	21:00			22:15		
51141353	11:00		12:30			13:30
51104634	8:00	9:30		10:40		
5545636813	17:00	18:00	19:00			
5527243387	11:00		12:00	13:15	14:00	15:00
5503623281	12:00			13:15		
5541759439	18:00		19:45		21:00	
5522604363		3:15		4:45		
5523448061				11:30		
5544799175			14:00	15:00		
5549179333		13:30		14:45		
5532291128	3:30				5:00	
5547276820	11:00	11:40		12:45		13:15
51165283			3:30		4:30	
5608655588			11:00			
5624990186				20:00		21:30
5517092137	5:30	7:00	8:00	9:00		
5549052324		4:30				
5528869553						16:15
5529950091	13:00		14:00			15:00
5543483009			4:00	5:00		6:00
5549963013	3:00	5:00		6:00	7:00	
5513793834	6:30	8:00	9:15	10:15		
5535707245						
5602759204	22:45					
5508137522			13:15	14:15		15:20
5529796107		3:30		4:30		5:30
5524291759			17:15			17:45
5617877359		4:00		5:45	6:45	
5535198704	21:30				23:00	
5508967072			10:30			11:30
5529673744						7:15
5533197975			4:45			
5507776093	4:00	5:20	6:30	7:30	8:30	9:30
5521734595	3:00	4:00	6:30	7:00	8:00	
51137841	8:30		10:00	11:00	12:00	13:00
5616167533	15:00	16:30	17:30		18:30	
5549787504	9:30		11:00			12:15
5625403549	4:00	5:00				
5607563229		2:00		3:00	4:00	

5543069444	20:30	21:15		22:30	23:15	
51219136	20:15					
5537391712	3:45	5:15	7:15			7:45
5538458207		6:30			7:30	8:30
5550116900	10:00	10:45	11:50		14:00	
51232707	21:30		22:45	23:45	0:30	1:30
5529753364	15:30		16:15	16:30		16:45
5545553755				22:15		
5548842863	2:45		3:45		5:00	
5624425768			9:30		11:00	12:00
5546111810					5:00	
5501318624						4:00
5616223208	2:30					3:30
5609356113	22:45	23:30	0:45	1:50		
5550726077	14:20		16:00	17:00	18:10	19:15
51257164	20:00	21:05			23:05	
5551175008	2:15					
5627973746						12:40
5553235852	5:30					
5507823583		9:15	10:45		11:45	12:45
5538705050	1:15			2:15		
5546585995		17:00		18:00	19:00	
51189799		2:30	3:15			
51096053			12:15	13:15		
5530148741	7:00	8:15	9:15	10:15	11:15	12:20
51293960	20:45					23:15
5523728553	21:15					
5625337669			14:30		16:00	
5538395559			11:00	12:00	13:00	14:00
5602659473	20:15		21:30	22:30		
51194087	21:00	22:10	23:15	0:15		1:15
5626254523	10:30			10:45		
51075017	13:45	15:15	16:30			17:35
5551168944	23:15	0:15		1:30		
5542991137						
5602738992			2:15		3:15	4:15
5552033557		15:30	16:45		17:45	18:45
51195927	21:00					
5623051503	6:30	7:30	8:00			8:50
5540689207	0:15		2:15	3:15	5:15	7:45
5554425720					8:30	9:30
5554775021				13:45		
51031031		2:30			3:15	

5624035445		21:45		23:45	1:15	2:15
5547574789		6:30	8:00		9:00	10:15
51143808	14:45	16:00	18:10	20:10	21:45	
5515877920		12:45				
51182428	4:00		5:30		6:45	
5554053278	2:45		4:00			
51254408		14:30	16:45	18:00	19:00	
51128988	5:30			6:30		7:30
5552302834	18:45			19:45		
51191709	21:00		22:15		23:15	
51140124	20h30		21h45		22h45	
5541865937	09h45	10h50	12h00	13H00	14h00	
51201995	21h45					
51185057	00h00					
5502659345	04H45				05h45	
51166114	02H15			03H30	04H30	
5536631472			05H35		06H15	07H15
5517838431	06H15					
5524523852				23H50		00H16
51052738	07H45					
5549722836			00H00			01H30
5512741685	09H30		11H30	13H30		
5506836106		14H55		16H05	17H00	18H15
5556858501			12H30			
5529796107		23H30		00H50		
5544286489			23H45			
51096600		06H15		07H00		08H05
5602373527			23H50	00H20		00H50
51283399	02H30	04H50	06H05		07H05	
5610891440	10H30			11H30		12H30
5539538240	00H30		02H00	03H00	04H00	05H00
5610132416			07H10	08H15	09H10	
5555325392	07H20	08H30	10H30	11H40	12H40	
5519948482	00H45	02H18	03H15			05H20
5557428777		15H00	16H00		18H00	
5556136657			13:00			
5544780583		04H00			04H30	
51176011	19H50	21H30		22H30	23H30	
5625060009	08H40	10H15	11H15		12H15	13H15
5531944655			22H10			23H10
12452888		21H15			22H15	23H15
5558830328	23H35		00H35		01H45	02H45
5516672310	16H15	17H15	19H15			20H15

5521098742					05H15	
5533004581			17H00		18H15	19H15
51208294	00H30		01H45			02H50
5550630996	07H50		09H00	10H00	11H00	12H00
5623288141	11H00	13H20	14H20	15H49		16H35
5558149510	11H05	11H30	12H30	13H30	15H40	16H40
5559844885			09H40		11H00	
5550434572			11H15		12H30	
5556265383	04H00		05H30	06H30	08H00	09H00
51223109				01H30		02H45
5532453604	21H50	22H50		23H50		00H50
5560222781	06H15	07H45	08H45	09H45		10H45
51064787	02H05		03H15			05H30
5560556928			05H45	06H45		07H10
5558571761	09H00		10H15	11H15	12H15	
5548803659		05H15	05H40			
5559035038	10H30		11H30			12H30
5627973746						04H11
5550088204	16H00		17H00	17H40	18H45	20H00
51068989	02H30	04H20	05H30			09H45
5560079103		21H20		22H50	23H50	00H50
5616595242			14H45			15H30
5561942614	17H45			18H50		
5547572567	22H30			23H40		00H50
51237783	14H00	15H30	16H30	17H30		22H00
5542044678		18H30		19H15		20H15
5560880462	07H00	11H00	12H00			13H15
5554900919	11H15			12H30	13H35	14H45
51123251		22H40				00H05
5560329279			06H30			08H00
5548125770	22H40		23H45		01H00	02H00
5563428936		09H45	11H15	12H15		
5533224449	15H30				17H15 LE 14/08/2017	
5504355532	02H30					
5613593090	11H15		12H50	00 le 22/09/2017		
5614812967		22H00		23H15	23H40	00H00
5545645604	07H00	09H15	11H10	12H10	15 LE 06/10/2	14H15
5559130422		05H30	06H30	08H00	09H20	12H20
51047336				06H10	07H25	
5502199304			07H45	09H20	10H45	
5563864729	11H00	13H15		20H00		21H00
51219136		20H15	21H30		22H30	02H25
5515803451		2:25				3:45

5563217354	9:15		11:00			12:00
51149551		20:00				
5548165075	9:15		11:00			
5542942940						17:15
5612955924	6:15		8:00	10:00		
5557894680	5:45					8:00
5518124276			4:45		5:20	5:55
51070794	15:30					17:00
5616355873		20:10	21:20		22:15	12:30
5553840484	3:15	5:15	7:15			8:15
5559461131	3:00	4:30	5:30	6:30		
5565462296	1:15		2:30	3:30	4:30	5:40
5623942485			22:00			
5613229544	7:50				8:55	11:00
5553682254			17:30		19:15	20:15
51236378			1:00			
5514651070	11:55			13:15	14:15	15:15
51185206			2:30			
5549727987		22:40		23:40		
5564256668		21:30	22:15	2:20		
5565872629		17:20	18:28	21:00	22:00	
5562475104			1:10			
5566290234					12:00	
5551323942			9:00		10:15	11:40
5625337669	7:45		9:15			10:45
5547625717			9:40		10:40	
5551259375	2:45			4:00		
5623051503						
5566738555						
5562096396	8:00			10:10	11:30	
5538768102	8:00	9:20	10:30		11:45	
5555092491	14:30	16:15	17:30	20:00	21:00	
51048621		5:30		7:00	8:00	10:00
5557998855	2:15	3:25				4:30
5541323343	1:45	3:45	4:45		7:45	
5524931353		13:00				14:40
5617529977			21:15			22:25
5566179591	10:50	12:00	13:00	14:00	15:20	16:20
5623940162		14:00		16:30		
5602738992	22:35		0:00	1:00	2:00	
5608180894				3:15		
5545014090		12:30	13:30	14:30		15:30
51103417	7:20	10:30	11:30	14:30	15:30	

5500415413	5:10	6:40	8:10	10:30	11:30	12:30
5610555273	5:00	6:15				
5532735308	22:50	0:45	2:00			3:15
51179056	21:30	22:40	23:40			
5502558507	3:50		5:00			6:00
22708911	18:15		20:45			

9 cm	10 cm	Ocytocine	entation du siège
	23:30	1	ège décomplété
	2:00	1	ège décomplété
	4:15	0	siège complet
	12:30	1	ège décomplété
	18:45	1	ège décomplété
20:00	20:30	1	ège décomplété
9:00	9:30	1	siège complet
	13:45	1	ège décomplété
10:15	11:00	1	ège décomplété
7:00	8:00	1	ège décomplété
	11:00	0	siège complet
0:45	1:00	1	siège complet
4:45	5:30	1	emi décomplété
15:30	16:30	1	ege décomplété
	5:30	1	ege décomplété
23:45	0:15	1	siège complet
	7:00	1	ège décomplété
1:15	2:15	0	ège décomplété
	6:15	1	ège décomplété
	18:15	1	ège décomplété
1:00	3:00	1	siège complet
10:15	10:40	1	ège décomplété
	4:15	1	ège décomplété
	7:15	1/1/1900 0:00	ège décomplété
4:30	5:00		siège décomplété
	4:15	1	ège décomplété
1:15	1:30	0	décomplété
5:00	6:00	1	décomplété
	22:30	1	décomplété
	12:30	1	décomplété
6:45	8:45	0	complet
11:45	12:00	0	complet
1:45	3:15	1	emi-décomplété
	23:00	1	ège décomplété
13:30	14:10	0	siège complet
	18:45	1	ege décomplété
	10:30	1	décomplété
	14:15	1	ege décomplété
	14:15	1	ege décomplété
	0:15	1	ege décomplété
	1:15	0	siège complet
	17:45	0	siège complet

	21:15	1	›mi décompleté
20:45	21:00	0	décompleté
20:15	21:15	1	décompleté
	9:30	1	décompleté
23:15	23:20	0	complet
	1:30	1	décompleté
10:30	11:30	1	décompleté
	8:30	1	décompleté
	3:30	0	complet
	11:15	1	décompleté
	12:00	1	décompleté
0:00	0:30	1	complet
20:00	21:00	1	décompleté
	21:00	1	décompleté
	3:45	1	décompleté
	8:15	1	complet
	10:20	1	décompleté
	15:15	1	complet
3:30	3:45	1	décompleté
2:15	3:00	1	décompleté
	12:30	1	décompleté
	15:45	0	décompleté
	16:45	1	complet
	4:15	1	complet
6:00	6:30	0	complet
	17:00	1	complet
	0:45	1	décompleté
	21:20	1	décompleté
1:15	2:00	1	décompleté
9:00	9:30	1	complet
	13:05	0	décompleté
	4:45	1	décompleté
	4:45	1	décompleté
7:45	8:45	1	décompleté
21:00	22:00	1	décompleté
3:30	14:15	1	décompleté
	22:00	1	complet
12:45	13h45	1	complet
	23:30	1	complet
	20:30	0	décompleté
22:45	23:10	0	décompleté
10:45	12:00	1	décompleté
	15:20	1	décompleté

0:50	1:15	0	complet	
	3:45	1	complet	
	23:30	1	complet	
	19:15	1	décompleté	
	4:00	1	décompleté	
7:00	7:15	1	décompleté	
	21:18	0	complet	
	9:45	1	complet	
19:45	21:00	1	complet	
3:30	4:30	1	décompleté	
	21:00	1	décompleté	
20:15	21:15	1	complet	
	11:00	1	complet	
	15:30	1	décompleté	
	5:30	1	décompleté	
	14:15	1	décompleté	
	1:40	1	complet	
8:00	9:00	1	décompleté	
	6:15	1	décompleté	
	17:15	1	complet	
	0:45	0	complet	
1:00	2:00	1	emi décompleté	
10:45	11:45	1	décompleté	
	17:15	1	décompleté	
	5:30	1	complet	
21:15	22:00	1	complet	
	2:30	1	complet	
10:00	11:00	1	décompleté	
	5:45	1	emi décompleté	
	21:30	1	décompleté	
	0:45	1	emi décompleté	
	5:45	1	décompleté	
	14:30	0	décompleté	
	19:15	1	décompleté	
	5:30	1	décompleté	
	17:00	1	emi décomplé	rétention tete derniere spatules
	22:40	1	décompleté	
	21:50	1	complet	
	6:15	0	complet	
21:00	22:30	1	emi décompleté	
	16:45	1	complet	
12:30	12:45	1	décompleté	
	10:30	1	complet	

10:00	11:15	1	complet	
	0:20	1	décomplété	
	8:00	0	décomplété	
23:09	0:30	1	décomplété	
	15:00	1	complet	
	11:10	1	décomplété	
	21:15	1	décomplété	
15:45	17:00	1	complet	
14:30	15:00	1	complet	
22:20	23:30	1	décomplété	
6:00	7:00	0	décomplété	
12:30	12:45	1	›mi décompléte	
15:45	17:00	1	décomplété	
	15:45	1	décomplété	
	5:45	0	décomplété	
13:30	14:00	1	›mi décompléte	
	5:30	1	décomplété	
12:15	13:15	1	décomplété	
22:45	23:45	1	décomplété	
10:00	11:00	1	décomplété	
5:45	6:30	1	décomplété	
	17:15	0	complet	procidence : grande extraction
17:00	18:00	1	décomplété	
7:00	9:00	1	décomplété	
	8:00	1	décomplété	
11:10	11:20	1	décomplété	
10:15	11:00	1	complet	
	0:15	0	décomplété	
16:15	19:20	1	décomplété	
	6:30	1	complet	
	18:45	0	complet	
8:00	9:00	1	décomplété	
	23:10	0	complet	
	11:45	1	complet	rétention tête denière
	8:45	1	décomplété	
	5:15	1	décomplété	
	10:00	1	décomplété	
8:20	9:30	1	complet	
	14:00	0	complet	
	19:45	0	décomplété	
13:15	14:45	1	décomplété	
5:45	6:00	0	décomplété	
	5:00	1	décomplété	

	0:15	1	décompléte
21:30	21:45	0	complet
	8:30	1	complet
	9:05	0	décompléte
15:00	15:30	1	décompléte
	2:45	1	complet
17:05	17:15	0	complet
	22:30	0	décompléte
	6:00	0	décompléte
13:00	14:00	1	décompléte
	5:30	0	complet
5:30	6:30	1	décompléte
	4:45	1	décompléte
	2:20	1	décompléte
	20:15	1	complet
	12:45	1	décompléte
	3:45	1	décompléte
	12:45	0	complet
6:15	7:30	1	décompléte
13:45	14:45	1	décompléte
2:45	3:30	1	complet
20:15	21:15	1	décompléte
	4:15	0	complet
	13:45	1	emi décompléte
12:40	12:45	1	décompléte
	0:40	1	emi décompléte
22:15	22:30	0	décompléte
17:00	18:05	1	décompléte
15:00	16:00	1	décompléte
	23:45	1	complet
	1:45	1	emi décompléte
	10:50	0	décompléte
18:35	19:45	1	décompléte
	3:00	1	emi décompléte
0:30	1:15	0	emi décompléte
	4:20	1	décompléte
19:45	21:45	1	décompléte
	22:30	0	décompléte
	9:45	1	décompléte
	8:45	1	décompléte
11:30	12:30	1	complet
	14:45	0	décompléte
	4:00	1	décompléte

3:30	5:30	1	décompléte
	11:30	1	décompléte
	22:50	1	complet
	14:30	1	décompléte
	8:30	1	décompléte
	5:00	1	décompléte
20:00	21:00	1	complet
8:30	8:58	1	décompléte
20:45	21:45	1	décompléte
0:30	1:30	0	décompléte
	00h58	1	siège complet
15h00	16h00	0	siège décompléte
22H45	23H45	1	siège complet
	01h10	0	siège décompléte
	06h45	1	siège décompléte
O7H00	09H00	1	siège décompléte
08H30	09H30	1	siège décompléte
	07H15	1	siège complet
	00H40	1	siège décompléte
	09H45	0	siège décompléte
02H30	03H30	1	siège complet
14H40	15H45	1	siège décompléte
	20H00	1	siège complet
14H15	15H30	1	siège décompléte
	02H00	0	siège complet
	00H45	0	siège décompléte
09H05	09H50	1	siège complet
	01H35	1	siège complet
	08H00	1	siège complet
13H30	15H30	1	siège complet
05H25	05H35	1	siège complet
09H19	09H28	1	siège décompléte
14H00	15h00	1	siège complet
06H30	07H35	1	siège complet
	21H25	1	siège décompléte
	13H45	0	siège décompléte
04H50	05H10	1	siège décompléte
	00H30	1	siège décompléte
14H45	15H45	1	siège décompléte
	01H00	1	siège complet
00H15	01h15	1	siège décompléte
05H30	08H50	1	siège complet
	21H15	1	siège décompléte

	06H20	1	siège décomplété	
19H45	20H45	1	siège décomplété	
03H50	04H50	1	siège décomplété	
13H00	14H00	1	siège complet	
17H30	18H45	1	siège décomplété	
17H50	18H50	1	siège décomplété	
	12H00	1	siège décomplété	
	13H30	1	siège décomplété	
10H00	11H00	1	siège décomplété	
03H45	07H55	1	siège décomplété	
01H50	02H55	1	siège décomplété	
11H45	12H45	1	siège décomplété	
06H10	06H29	1	siège complet	
07H22	07H37	1	siège complet	
13H15	14H40	1	siège décomplété	
	06H10	1	siège complet	
	13H30	1	siège décomplété	
	04H19	0	siège décomplété	
22H20	23H00	1	siège complet	
	10H15	1	siège décomplété	
01H50	03H00	1	siège décomplété	
15H37	16H03	1	siège complet	
19H50	20H50	1	siège décomplété	
	01H50	0	siège complet	
	00H00	1	siège décomplété	
21H15	22H45	0	siège décomplété	
14H24	15H16	1	siège décomplété	
16H30	18H15	1	siège décomplété	
	00H56	1	siège décomplété	
10H15	11H10	1	siège complet	
	04H45	1	siège décomplété	
13H15	15H00	0	siège décomplété	
18H25	18H30	0	siège décomplété	
03H50	05H00	0	siège complet	
15H00	16H20	0	siège décomplété	
02H45	02H53	0	siège complet	
15H15	15H50	1	siège complet	
	13H10	1	siège décomplété	
08H30	09H30	1	siège complet	
12H45	13H45	1	siège décomplété	
22H00	00H00	1		siège décomplété
	02H35	1		siège décomplété
	4:00	0	complet	siège complet

	13:30	0	décompléte	siège décompléte
21:30	22:30	0	complet	siège complet
	13:20	1	complet	siège décompléte
	17:38	0	complet	siège décompléte
	10:50	1	décompléte	siège complet
9:00	10:00	1	décompléte	siège complet
	7:00	0	complet	
	18:15	1	décompléte	
2:55	4:55	1	complet	
9:15	10:15	1	complet	
	7:45	1	décompléte	
9:15	10:15	1	décompléte	
23:15	0:00	0	décompléte	
12:00	14:05	1	décompléte	
21:15	22:15	0	complet	
	2:30	0	complet	
16:15	17:15	0	décompléte	
3:45	5:00	0	complet	
	1:25	1	décompléte	
	3:10	1	complet	
	23:00	1	ɛmi-décompléte	
	3:00	0	décompléte	
	13:30	1	complet	
15:00	16:10	1	décompléte	
	11:30	0	décompléte	
	12:00	1	complet	
	5:10	0	complet	
6:45	6:55	0	ɛmi decompléte	
15:40	15:50	0	complet	
14:00	15:00	1	complet	
12:40	13:00	1	décompléte	
22:00	0:15	1	complet	
12:30	13:30	1	décompléte	
	5:20	0	décompléte	
	6:45	1	décompléte	
15:50	17:20	1	ɛmi décompléte	
23:25	0:25	0	décompléte	
18:10	20:00	1	décompléte	
	17:30	1	complet	
3:00	3:50	1	décompléte	
	4:40	1	décompléte	
16:30	16:53	0	ɛmi décompléte	
	17:10	1	complet	

14:30	17:30	1	décomplété
	6:20	0	emi décomplété
	4:30	1	complet
	0:40	0	complet
7:00	8:00	1	décomplété
21:45	22:45	1	décomplété
