# Supplementary material for: Analysis of spontaneous labor progression of breech presentation at term
Source: PLoS One. 2022 Mar 14;17(3):e0262002. doi: 10.1371/journal.pone.0262002 (PMC8920216; doi:10.1371/journal.pone.0262002)
Supplement: S2 File — (PDF) [file pone.0262002.s003.pdf]

age	gestational age	BMI	gestity	parity	epidural analgesia
26 years	37.6	25	1	0	1
32 years	41	24	3	1	0
36 years	40	34	3	1	0
31 years	38	37	4	2	1
40 years	38.6	19	5	3	1
36 years	39.5	25	1	0	1
37 years	39.1	22	4	2	1
29 years	39.6	25	1	0	1
30 years	39.4	19	3	1	1
29 years	40.1	25	1	0	1
31 years	39.5	28	4	2	1
40 years	41.4	24	6	5	1
26 years	39	32	1	0	1
34 years	39.6	20	2	0	1
31 years	40.5	20	2	1	1
33 years	40	18	3	1	1
26 years	41.3	21	2	0	1
30 years	38.6	20	2	0	1
27 years	39.6	21	3	0	1
28 years	40.4	29	3	2	1
31 years	40.1	19	3	1	1
32 years	39.5	24	1	0	1
21 years	41.2	23	1	0	1
22 years	40.1	18	1	0	1
42 years	41.3	18	8	5	1
33 years	40.5	23	1	0	1
32 years	40.6	19	1	0	1
31 years	39.5	22	2	1	1
42 years	40.3	27	8	6	1
24 years	39.5	29	1	0	1
41 years	37	18	4	2	1
37 years	40.6	31	3	2	1
37 years	41.3	28	2	1	1
25 years	38.5	20	1	0	1
28 years	40.5	23	2	0	1
42 years	39.4	19	4	1	1
42 years	38.3	25	8	3	1
27 years	38.4	20	1	0	1
28 years	41.5	21	1	0	1
34 years	40	27	2	0	1
29 years	40.2	22	3	1	1
35 years	41.3	30	6	4	0

24 years	38.3	24	3	2	1
36 years	41.1	19	2	1	1
29 years	39	20	1	0	1
32 years	40.6	22	2	1	1
27 years	39.1	23	2	1	1
34 years	39.1	31	2	1	0
28 years	40.1	24	1	0	1
26 years	40.1	19	2	1	1
28 years	40.1	20	5	2	1
28 years	40.6	33	8	6	0
41 years	39.4	27	2	1	1
33 years	39.2	21	2	1	1
23 years	39.5	19	1	0	1
31 years	38.6	18	1	0	1
30 years	39.5	24	1	0	1
30 years	39.3	21	1	0	1
31 years	37.6	20	3	2	1
30 years	39.5	19	3	1	1
33 years	40.3	22	2	1	1
33 years	40.3	21	1	0	1
29 years	39.2	21	1	0	1
33 years	39.2	20	1	0	1
33 years	39	21	4	3	1
22 years	39.4	22	4	1	1
26 years	40.4	32	2	1	1
27 years	40	18	2	1	1
25 years	40.3	17	1	0	1
33 years	38.3	19	1	0	1
29 years	37.2	25	1	0	1
26 years	38.3	21	3	1	1
24 years	37.2	30	1	0	1
27 years	38.1	20	2	1	1
19 years	39	24	1	0	1
30 years	39.2	22	1	0	1
30 years	40.1	19	1	0	1
33 years	41.1	24	1	0	1
30 years	37.5	18	2	0	1
29 years	39.2	22	2	1	1
26 years	41.1	0	4	2	1
37 years	39.2	29	2	0	1
25 years	39.5	18	1	0	1
34 years	41.1	26	4	2	1
32 years	39.6	16	1	0	1

26 years	38.2	25	1	0	1
34 years	40.1	21	3	2	1
41 years	40.6	21	3	1	1
36 years	39.1	35	4	2	1
34 years	41.3	19	2	1	1
34 years	38.3	18	2	0	1
41 years	39.1	20	2	1	1
40 years	38.5	19	5	2	1
28 years	41.2	23	1	0	1
27 years	40.1	27	1	0	1
27 years	40.5	22	1	0	1
36 years	40.3	22	3	2	1
32 years	39.4	22	1	0	1
29 years	40.6	22	2	1	1
31 years	38.6	21	1	0	1
36 years	40.5	20	3	0	1
31 years	40.4	22	2	0	1
30 years	40.6	40	2	1	1
31 years	39.3	19	1	0	1
28 years	40.4	24	1	0	1
22 years	39.2	35	1	0	1
28 years	39.2	24	4	3	1
27 years	40.1	19	1	0	1
27 years	41.1	21	2	1	1
31 years	37.6	18	1	0	1
29 years	40	21	2	1	1
28 years	40	36	1	0	1
29 years	40	20	1	0	1
37 years	39	35	1	0	1
28 years	38.1	19	1	0	1
20 years	38.4	22	1	0	1
31 years	41.1	40	2	1	1
35 years	40.1	17	3	2	1
28 years	40.5	20	5	2	0
29 years	37.1	25	3	1	1
26 years	39.2	19	5	4	1
35 years	40.6	23	4	1	1
34 years	37.2	18	2	1	1
39 years	41.1	22	6	1	1
35 years	41.1	23	1	0	1
36 years	37.5	31	6	5	1
32 years	39.1	36	4	1	1
31 years	39	26	2	1	1

27 years	38.1	21	1	0	1
34 years	40.1	24	2	0	1
33 years	37.1	32	8	7	1
30 years	39.4	29	1	0	1
28 years	39.1	19	1	0	1
33 years	39.5	21	2	0	1
24 years	39.3	23	1	0	1
36 years	39.2	19	3	1	1
27 years	40.3	29	2	0	1
32 years	41.1	27	1	0	1
36 years	37.2	22	2	0	1
42 years	39.6	25	4	3	1
23 years	40.5	26	1	0	1
30 years	40.2	22	1	0	1
32 years	40.1	20	2	1	1
31 years	40.6	21	1	0	1
26 years	37.2	25	1	0	1
32 years	40.1	23	4	3	1
28 years	40.2	31	3	2	1
29 years	39.6	19	1	0	1
26 years	40.4	20	1	0	1
34 years	40.4	20	2	1	1
28 years	40.6	27	1	0	1
35 years	40.1	23	1	0	1
28 years	37.2	24	6	4	1
26 years	40.2	29	1	0	0
33 years	39.5	25	2	1	1
39 years	38.4	23	3	2	1
28 years	40.5	21	2	1	1
19 years	38	18	2	0	1
24 years	37.6	20	2	1	1
25 years	40.4	22	1	0	1
24 years	39.5	19	4	1	1
27 years	39.3	21	2	1	1
32 years	40.2	22	4	0	1
25 years	38.5	25	1	0	1
28 years	40.6	17	1	0	1
30 years	38.2	19	6	2	1
31 years	38.1	22	2	0	1
36 years	39.3	20	1	0	1
29 years	39.4	20	1	0	1
26 years	39.2	34	1	0	1
19 years	39.5	25	4	0	1

30 years	37.4	23	1	0	1
25 years	40.5	24	2	2	1
24 years	39.6	19	2	1	1
33 years	38.6	19	2	0	1
26 years	40.3	21	1	0	1
20 years	41.1	36	2	0	1
31 years	38.6	21	2	0	1
32 years	40.2	19	5	4	1
26 years	38.5	21	6	3	0
38 years	38.3	25	4	2	1
32 years	39.1	21	1	0	1
39 years	40.2	35	8	4	0
39 years	41.4	21	5	2	1
37 years	38.1	24	2	1	1
26 years	41.3	28	2	1	1
28 years	39.2	27	3	0	1
28 years	40.4	19	1	0	1
26 years	40.1	20	1	0	1
27 years	39.6	21	1	0	1
39 years	39.1	18	1	0	1
32 years	39.1	20	2	1	1
30 years	39.2	21	1	0	1
35 years	40.6	24	3	0	1
28 years	41	20	1	0	1
32 years	39.4	19	3	1	1
27 years	38.4	29	2	1	1
35 years	39.6	23	5	3	1
31 years	37.3	21	1	0	1
21 years	39.1	24	4	1	1
29 years	40.1	25	5	4	1
27 years	41	20	1	0	1
31 years	41.4	19	2	1	1
36 years	40.1	16	2	1	0
29 years	38.5	21	1	0	1
20 years	38.3	18	1	0	1
23 years	40.3	21	3	0	1
33 years	38.1	23	1	0	1
22 years	40.6	24	1	0	1
29 years	41.1	20	2	1	1
28 years	41	20	2	0	1
30 years	38.2	18	1	0	1
30 years	40.4	21	2	1	1
37 years	41.6	24	3	2	1

35 years	37.1	18	3	1	1
32 years	41.2	22	3	1	0
38 years	41.1	23	1	0	1
29 years	39	18	1	0	1
30 years	39.2	20	2	1	1
32 years	39.3	22	3	0	1
20 years	39.2	26	1	0	1
20 years	39	28	2	0	1
25 years	40.3	23	2	0	1
25 years	40.3	20	2	0	1
32 years	39.3	22	1	0	1
36 years	39.4	18	1	0	1
35 years	39.6	17	3	1	1
29 years	38.3	20	2	0	1
28 years	39.6	23	1	0	1
26 years	38	30	1	0	1
33 years	40.6	20	3	1	1
30 years	41.1	24	1	0	1
31 years	39.2	21	2	1	1
22 years	37.4	20	2	1	1
29 years	39.3	22	2	1	1
33 years	37.4	19	1	0	1
24 years	38.6	20	1	0	1
30 years	40.2	18	1	0	1
20 years	38.2	19	2	1	1
24 years	38.3	23	2	1	1
28 years	41	22	3	1	1
30 years	41	28	4	3	1
33 years	38.3	30	1	0	1
29 years	40.2	18	2	1	1
30 years	38.1	17	7	2	1
37 years	38.2	19	2	1	1
27 years	40.2	19	1	0	1
31 years	39.4	29	7	5	1
31 years	39.1	22	1	0	1
33 years	39.3	23	2	1	1
26 years	40	20	3	2	1
28 years	39	21	1	0	1
27 years	40.6	23	1	0	1
33 years	39.5	24	4	3	1
33 years	38.4	24	3	1	1
36 years	39.6	25	3	2	1
28 years	38.1	22	2	0	1

34 years	39.1	27	2	1	1
28 years	40	17	2	1	1
32 years	41	21	3	0	1
34 years	38	25	1	0	1
27 years	39.5	22	1	0	1
33 years	39.1	21	1	0	1
25 years	37.2	22	1	0	1
30 years	39.5	20	1	0	1
27 years	40.6	20	1	0	1
26 years	38	19	1	0	1
33 years	41.1	26	1	0	1
26 years	39.3	26	1	0	1
39 years	41	24	1	0	1
33 years	39	25	3	2	1
33 years	40.4	21	2	1	1
28 years	40.5	21	1	0	1
23 years	39.4	25	1	0	1
29 years	39.6	21	2	1	0
33 years	39.6	23	2	0	1
22 years	40.3	21	2	1	1
31 years	38.6	21	1	0	1
27 years	38.5	22	2	1	1
30 years	37.3	18	1	0	1
38 years	39.6	20	5	1	1
30 years	39.2	30	7	3	1
32 years	39.2	27	3	1	1
27 years	38.1	32	1	0	1
29 years	41.1	23	2	1	1
28 years	40.2	18	1	0	1
34 years	39.3	25	2	0	1
26 years	39	23	1	0	1
34 years	38	17	4	2	1
29 years	39.2	20	6	1	1
24 years	38.2	23	1	0	1
28 years	39	25	1	0	1
39 years	39	24	4	2	1
33 years	40.5	24	5	4	1
19 years	38.5	23	1	0	1
35 years	39.4	22	5	2	1
27 years	39.4	22	1	0	1
27 years	39	22	1	0	1
35 years	41	31	2	0	1
30 years	37.5	19	4	0	1

31 years	37.3	27	2	1	1
32 years	37.3	26	2	1	1
34 years	39.6	19	2	1	0
27 years	37.3	22	1	0	1
28 years	40.4	23	1	0	1
34 years	39.2	19	3	1	1
33 years	37	24	2	0	1
32 years	39.5	23	2	0	1
32 years	39	0	2	1	1
33 years	37.3	23	4	2	1
33 years	41.1	21	1	0	1
24 years	40	21	2	0	1
20 years	41	36	3	1	1
35 years	38.3	23	3	1	1
36 years	40	19	1	0	1
34 years	41.3	18	3	2	1
44 years	38.6	28	4	3	1
21 years	40.2	19	3	2	1
36 years	40.1	34	2	1	1
24 years	39.1	17	1	0	1
32 years	37.1	21	1	0	1
15 years	39.1	0	1	0	1
31 years	37.3	25	3	2	1
24 years	39.5	28	6	4	1
29 years	38.4	23	1	0	1
30 years	40	22	2	1	1
31 years	39.5	20	3	1	0
31 years	40.3	21	3	2	0
30 years	40.5	18	2	1	1
31 years	38	30	3	1	1
28 years	37.6	27	2	0	1
34 years	40.6	20	5	1	1
34 years	38.5	24	1	0	1
27 years	39.6	19	3	1	1
36 years	41.3	21	2	1	1
34 years	37.1	20	2	1	1
32 years	41.1	25	1	0	1
26 years	40.1	27	6	2	1
36 years	41.1	24	2	1	1
35 years	39.4	36	2	1	1
29 years	40.5	21	1	0	1
30 years	37.1	23	1	0	1
34 years	39.5	19	2	0	1

30 years	39.6	19	3	1	0
35 years	41.4	24	2	1	1
30 years	40.3	20	1	0	1
35 years	40.3	31	2	0	1
27 years	37	26	2	0	1

breech presentation	episiotomy	Birth injury perineum	n bith weight	lead circumfe
franck	0	First degree	3260	36.5
franck	0		3000	0
complete	0		3800	34
franck	0		3030	34.5
franck	0	Second Degree	3130	35
franck	0	First degree	3030	36
complete	0		3240	33.5
franck	0		3310	33.5
franck	0	First degree	3220	35
franck	0	First degree	3290	35
complete	0		3400	31
complete	0		2260	32
franck	0	First degree	2950	36
franck	0		3020	34.5
franck	0		3350	33
franck	1		2550	33.5
franck	0	Second Degree	3320	34
franck	0		2610	33.5
franck	0	First degree	3340	34.5
franck	0		3650	35.5
complete	0	First degree	2830	34
franck	1		2680	34
franck	0	Second Degree	3100	34
franck	0		2870	34
franck	0		2790	34
franck	1		3070	35
franck	0	First degree	3100	36
franck	0		3070	34
franck	0		3270	33.5
franck	0		2930	35
franck	0		2510	31
complete	0		3710	37
franck	0	First degree	3440	35
franck	0		2500	34
franck	0		2870	34.5
complete	0	First degree	2930	33
franck	0		2950	30
franck	0		2750	34
franck	0	Second Degree	3710	37.5
complete	0		2940	34
franck	0		3040	35
complete	0		3280	34

complete	0		3010	34
franck	0	Second Degree	3360	33.5
franck	0		2760	34
franck	0		3380	36.5
franck	0		3320	36
complete	0	First degree	2750	34.5
franck	0	First degree	3370	35.5
franck	0	Second Degree	3430	34
franck	0	Third Degree	3680	36
complete	0		3030	35
franck	0	Second Degree	3540	35
franck	0		3120	35.5
complete	0		2340	32.5
franck	1		3200	35.5
franck	1		2940	0
complete	1	Second Degree	3020	35
complete	0	First degree	3150	35.5
franck	0		2980	35
complete	0		2310	32
franck	1		2750	33
franck	0		3290	36
franck	0	Second Degree	2980	33
franck	0		2860	34.5
complete	0		3080	34.5
complete	0	First degree	3660	37
complete	0		2850	33
complete	0	First degree	2860	33
franck	0		3010	35
franck	1		2320	32.5
franck	0		2990	32
complete	0	First degree	3770	35
franck	0		2950	37
franck	0		2510	34
franck	1		3760	36
franck	1		3040	36
franck	0	Second Degree	3240	36
franck	0		2820	33
complete	0		3070	34
complete	0		3020	35
complete	0	First degree	3115	33
franck	0		2785	33
franck	0		3150	34.5
franck	0		3190	33

franck	0		2700	34.5
complete	0		3200	35
complete	0		3115	34.5
franck	0		2870	34
franck	0		3075	35
franck	0		2990	35.5
franck	0		3025	34
complete	0	First degree	3150	34
complete	0	First degree	3510	35.5
complete	0		3400	36
franck	0	First degree	3130	36
franck	0		3280	35
complete	0		3050	35
complete	0		4000	35
franck	0		3015	34.5
franck	0	First degree	3125	34.5
complete	0		3150	0
complete	0		3760	35
franck	0	First degree	2880	34
franck	0		4100	39
complete	0		3400	35.5
complete	0		3200	33
complete	0		4090	35
franck	1		3140	34.5
franck	1		3070	34
franck	0		2680	33
complete	0		3035	33.5
complete	0		3075	35
franck	0		2570	31
complete	0		2150	32
franck	0		2395	32
franck	0	First degree	3600	37
franck	0		2870	35
franck	0		3300	35.5
franck	0		2900	35
franck	0		3390	37
complete	0		3890	39
franck	0		3070	35
complete	0		3125	34
franck	0		3440	36
complete	0		2945	33
franck	0		2990	36
complete	0		3210	35

complete	0		3060	33.5
franck	0	Second Degree	3410	35.5
franck	0		2590	33
franck	0		3760	37
complete	0	Second Degree	2650	33
franck	0		2650	33
franck	1		3600	34
complete	0		3160	35
complete	0		2880	34.5
franck	0	Second Degree	2700	33
franck	0		2460	37
franck	0		3770	36
franck	0		3090	34
franck	0	First degree	2740	34.5
franck	0		3340	36
franck	0		2770	34.5
franck	0	First degree	2330	31
franck	0		2780	33.5
franck	0		3280	34
franck	0	First degree	3620	36
franck	0		2910	35
complete	0		3820	35
franck	0	First degree	3260	37
franck	0		2910	35
franck	0		3300	34
franck	0	First degree	2980	34.5
complete	0		2870	33.5
complete	0		2860	32
franck	0		3650	36
complete	0		2450	33
complete	0		3080	33
franck	0	Second Degree	2830	34
complete	0		3370	34
complete	0		2250	32
franck	0		2620	33.5
franck	0		3130	35
franck	1		2600	34
franck	0		2400	33.5
complete	0		2780	34
complete	0		3015	33
franck	0		2890	35
franck	0	First degree	3100	34.5
franck	0	First degree	2490	0

franck	1		2620	33.5
franck	0	Second Degree	3700	35.5
complete	0		2800	35.5
complete	0		3080	35
franck	0		3230	34.5
franck	0		3640	36.5
complete	0		3260	35
complete	0		2790	36
franck	0		3410	34
franck	0		3260	36.5
franck	0		3710	36.5
complete	0		3280	35
franck	0		4310	38.5
franck	0		2280	32.5
franck	0		3260	35.5
complete	0		3170	34
franck	0		2960	35
franck	0	Second Degree	3280	35.5
complete	0		2650	34
franck	1	Third Degree	2680	35
franck	0		3560	36
complete	0	First degree	2740	33
franck	0		2870	34.5
complete	0		3310	33
complete	0		2750	34
franck	0		3050	35.5
complete	0		4000	36.5
franck	0		3150	34
franck	0		3140	33.5
franck	0		3490	35
complete	0		3490	34
franck	0		3220	36
franck	0		2745	33
franck	0	First degree	3310	35
franck	0		2460	32
franck	0		2660	34
franck	0		3070	33
franck	0		3490	33.5
franck	0		3720	36.5
franck	0	Second Degree	2770	34
franck	0		2520	33
complete	0		3740	35
franck	0		3490	37

franck	0		2540	33
franck	0	First degree	4090	37
franck	0		3470	36.5
complete	0	Second Degree	2880	36
franck	0	Third Degree	3130	34.5
franck	1		2800	36
franck	0		2930	33.5
complete	0		3870	36
franck	1	Fourth Degree	2670	33
franck	0		3700	0
franck	0	Second Degree	2690	34
franck	0		2950	34.5
complete	0		2440	34
franck	0		2310	34.5
franck	0		3140	33.5
franck	0	Second Degree	3350	35
franck	0	First degree	3240	35.5
franck	0		3250	35.5
complete	0		3430	34
franck	0		2430	31.5
complete	0		3430	34
franck	0		2960	34.5
complete	0		2560	35
franck	0	Second Degree	3700	35
complete	0		2880	33
complete	0		2350	33
complete	0		3850	36
complete	0	First degree	3325	36
complete	0		2700	34
franck	0	First degree	4020	36
complete	0		2500	32
complete	0	First degree	3070	34
franck	1		3320	34.5
franck	0		3080	36
franck	0		3045	34.5
franck	0	First degree	3150	36
franck	0		3290	34
complete	0	First degree	2400	33
franck	0		3480	35.5
complete	0		2790	33
franck	0		3680	36
franck	0		3575	36
franck	0	Second Degree	2880	34

franck	0		3100	34
complete	0		3540	34.5
franck	0		3290	33
franck	0		2770	34
franck	1	Third Degree	3120	34
franck	0		2765	35
franck	0		2335	32
franck	0	First degree	2970	33.5
franck	0		3680	35.5
franck	0		2880	34
complete	0		4190	36
complete	0	First degree	3110	35
franck	1		2520	34
complete	0		2760	36
franck	0	First degree	3050	34
franck	0		2490	33.5
franck	0	First degree	2970	34
complete	0		3400	33.5
franck	0	Third Degree	3780	37
franck	0		3290	35
franck	0	Second Degree	3270	34
franck	0		2545	33
franck	0		2600	32
franck	0		3780	36
complete	0		3000	35
franck	0		3290	35.5
franck	0		2690	34
franck	0		4200	36.5
franck	0		3875	34.5
complete	0		3020	35.5
complete	0		3390	35
franck	0		3180	36
complete	0		3150	31.5
franck	0		3070	32.5
franck	0		2630	32.5
complete	0	Second Degree	3070	35
franck	0		3650	36
complete	0		2360	30
franck	0		3350	36
franck	0		2860	34
complete	0		2990	34
complete	0	First degree	3500	36
franck	0		2500	31

complete	0		2490	33
complete	0		2420	32.5
complete	0		2660	34
franck	0		2210	33.5
franck	0		3220	35.5
complete	0		2770	32.5
franck	0		2400	32.5
franck	1	Second Degree	3470	35
complete	0		3900	35.5
franck	0		2570	33
franck	0		3590	36
franck	0		2750	33
franck	0		3490	34
complete	0	Second Degree	3310	33.5
complete	0		2890	33
franck	0	First degree	4100	37
complete	0		3210	33
franck	0		3280	35
complete	0		2620	32
franck	1		3500	35
franck	0		2745	34
franck	0		2750	34
franck	0		3155	36
franck	0		3320	34
franck	0	First degree	3110	36
complete	0	Third Degree	3060	34
complete	0		2180	32
complete	0		3130	35
complete	0		4260	36
franck	0		3420	33
complete	0		2890	33.5
franck	0		3070	33.5
franck	0		2640	33.5
franck	0		3520	35
franck	0		2980	34.5
franck	0		2960	35
franck	0		3170	34
complete	0		3310	33
franck	0		3400	34
franck	0		3380	35
complete	0		3390	34
complete	0		2650	32
franck	0		3110	34

franck	0		3000	33.5
franck	0		3600	33.5
complete	0		2760	33
franck	0		3590	35
franck	0	First degree	2720	34

Apgar score	bilical arterial	reterial lactat	Newborn transfer	ostpartum hemorrhage (ml)
10	7.2	NC		10
4	6.99	NC	onatal intensive care u	100
10	7.15	NC		750
10	7.14	NC		50
10	7.17	NC		50
10	6.82	6.25		200
10	7.25	NC		150
10	7.12	NC		100
10	7.27	NC		150
10	7.31	NC		100
10	0	NC		20
10	7.14	7.21		100
10	7.12	7.94		50
10	7.33	3.2		50
10	7.25	2.87		30
10	7.05	11.4		200
10	7.22	4.4		150
10	7.12	6.85		50
10	7.16	7.45		250
10	7.33	2.02		800
10	0	3.9		50
10	7.12	9.5		100
9	7.01	9.41	onatal intensive care u	100
10	7.15	7.5		250
10	7.102	2.15		0
10	7.12	5.2		300
9	7.14	8.92		150
10	7.15	4.84		250
7	6.93	5.76	onatal intensive care u	50
10	7.22	5.63		50
10	7.29	3		50
10	7.24	3.61	neonatal unit	450
10	7.29	3.11		250
10	7.16	6.88		250
10	7.19	8.72		50
9	7.04	6.63		50
10	7.09	7.64		100
10	7.23	5.13		100
10	7.16	7.16		300
10	7.08	5		50
10	0	5		100
10	6.965	5.65		200

10	7.22	7.64		50
10	7.29	6.19		100
9	7.046	10.78		100
10	7.29	2.89		400
10	0	5.2		50
10	7.26	2.68		100
10	7.08	7.8		50
10	7.33	3.85		350
10	7.25	3.15		150
10	7.32	3.28		100
9	7.03	5.91	neonatal unit	100
10	6.87	13		100
10	7.13	10		100
10	7.01	10		200
6	7.05	11	neonatal unit	1100
10	7.41	7.68		100
8	7.21	2.34		150
10	0	4		50
10	7.11	9.62		100
10	7.16	7.38		50
10	0	5.1		100
10	0	9.1		750
10	7.31	4.98		50
10	7.18	4.03		250
10	7.32	5.38		100
10	7.11	5		150
10	7.28	6.1		50
9	7.24	6.72		50
10	7.1	7.43		100
10	7.246	5.75		400
10	7.19	6.12		750
9	7.09	8.82		50
10	0	0		50
10	6.95	6.58		100
10	7.09	11.33		200
10	7.15	7.91		250
10	7.35	4.08		50
10	7.32	2.95		50
10	7.14	4.72		50
10	0	4.1		400
10	0	5.93		50
10	7.19	5.41		20
10	7.21	7.94		250

9	6.81	13		50
10	0	3.6		200
10	7.14	6.39		300
10	7.27	2.8		150
10	0	3.98		300
10	7.19	3.63		150
10	7.24	3.46		50
10	7.17	6.16		100
10	6.84	7.06		50
10	7.23	4.76		150
10	7.24	3.93		50
10	7.22	3.98		50
10	7.29	5.66		250
10	7.26	2.51		200
10	6.86	8.48		50
10	7.27	2.95		50
8	6.9	6.7		50
10	7.13	6.2		100
10	7.16	6.59		150
9	6.96	6.48		350
10	7.24	3.23		100
10	7.09	7.44		50
10	7.34	3.21		100
10	7.07	6.85		50
10	7.17	7.05		200
10	0	5.8		100
10	7.323	1.93		50
10	7.11	6.8		50
10	7.19	5.76		100
10	7.04	11.94		100
10	6.99	9	neonatal unit	50
10	7.16	5.97		50
10	0	2.9		50
10	7.21	4.32		50
9	7.3	5.1	neonatal unit	50
10	0	3.8		450
9	0	4.6		300
10	7.17	5.06		100
9	7.053	8.37		150
10	7.023	8.43		50
10	7.31	3.06		50
8	7.12	6.7		150
9	7.185	3.75		50

10	7.32	3.58		200
10	7.07	7.15		50
10	7.23	4.1		100
10	7.237	3.05		100
10	7.26	4.75		50
10	7.18	5.76		50
10	7.25	5.45		50
9	6.96	9.1		50
10	7.15	7.19		100
10	6.89	13.59	neonatal unit	200
10	7.18	6.42		100
10	7.1	4.5		70
10	7.21	6.46		50
10	7.09	7.77		100
10	0	5.8		150
9	6.94	10		50
10	7.24	3.66		200
10	7.29	2.49		50
10	0	4		100
10	7.19	4.56		800
10	6.87	12		150
10	7.21	3.29		250
10	7.06	9.24		250
10	0	4		500
8	7.18	4.71		250
10	7.07	8.45		50
10	7.13	6		50
10	0	5.2		50
10	7.04	8.89		50
10	0	6		50
10	7.32	3.28		300
9	6.99	9		50
10	7.2	2.92		50
7	7.33	3.42		200
9	7.03	8		50
10	7.25	3.7		400
10	7.04	9.66		100
10	7.35	3.9		100
10	7.01	8.38		20
10	7.33	3.31		300
10	7.22	3.53		100
10	7.05	8.54		100
10	7.14	5.54		50

10	7.011	7	50
10	7.3	2.72	50
9	7.2	6.69	50
10	7.35	1.73	350
10	6.93	9.82	50
10	7.37	4.15	200
10	7.08	11.19	150
10	7.22	7.22	150
10	7.29	3.52	50
10	7.13	7.06	100
10	7.11	7.39	200
10	7.1	6.32	50
10	7.08	8.24	50
10	7.133	6.92	50
10	7.17	5.6	200
10	7.01	10.2	800
10	7.22	4.65	150
10	7.22	5.09	50
10	7.23	6.66	50
10	7	8.5	50
10	7.02	7.27	50
10	7.31	4.17	50
10	7.16	5.78	500
10	7.33	3.26	100
10	7.21	3.02	50
9	7.02	7.79	200
10	7.15	5.77	150
10	7.19	5.91	20
10	7.29	2.11	50
10	7.01	6.68	50
10	7.32	3.9	200
10	7.198	3.31	150
10	7.23	5	20
10	7.13	4.75	50
10	7.17	4.9	50
10	7.24	2.33	25
10	7.2	5.96	250
10	0	3.7	400
9	6.98	10.6	250
10	6.86	5.58	150
10	7.18	7.27	100
10	7.21	5.19	350
10	7.1	6.2	50

10	7.37	3.18	50
10	7.13	5.02	450
10	7.03	7.9	100
10	7.05	6.58	150
9	7.08	6.5	200
10	7.27	4.25	400
10	6.99	8.11	50
10	7.31	3.29	50
8	6.86	5.89	150
10	6.97	8.24	50
10	7.05	9.37	50
10	7.23	3.48	200
10	7.28	3.91	50
10	7.22	3.33	100
10	7.08	6.92	50
10	7.23	4.35	200
10	7.19	3.33	50
9	7.07	8.56	50
10	7.2	8.38	50
9	7.14	4.84	50
10	nc	3.43	50
10	7.01	9.85	150
10	7.18	4.73	150
10	7.08	6.89	50
10	7.21	4.6	100
10	7.03	6.85	200
10	7.18	3.5	100
10	7.26	2.16	50
10	7.19	4.9	150
9	7.28	2.06	200
10	7.18	3.64	150
10	7.1	5.97	200
8	6.84	6.17	50
10	7.15	6.87	50
9	7.085	5.78	50
10	7.17	5.91	50
10	7.32	1.92	200
10	7.01	11.58	250
10	6.93	11.18	50
10	7.06	7.13	350
10	7.06	7.05	500
10	7.09	8.56	50
10	7.3	4.14	50

10	7.11	8.34		50
10	7.072	9.69		50
10	7.13	5.2		250
10	7.18	2.77		50
10	nc	5.3		600
7	7.01	8.75		100
10	7.19	6.25		300
10	7.04	7.66		50
6	6.8	10.9		50
10	7.19	5.12		50
10	7.187	5.29		100
10	7.09	6.17		50
6	7.005	10.27		800
9	7.09	6.74		100
10	7.08	6.81		50
10	0	5.6		40
10	0	5.6		50
10	7.3	2.89		50
10	7.042	7.87		200
10	7.176	5.86		100
10	7.11	5.96		50
10	7.12	6.79		50
9	7.23	6.54		100
10	7.35	2.68		50
6	7.21	4.24	onatal intensive care u	750
10	7.024	7.44		50
10	7.14	6.02		50
10	7.14	6.11		150
9	7.18	6.36		250
10	7.24	3.57		100
8	7.2	8.42		50
10	7.231	3.25		25
9	7.17	5		50
10	7.24	2.73		150
10	7.16	4.56		50
10	7.146	6.59		100
8	7	7.9		50
7	7.31	5.7		150
10	7.25	5		50
10	7.14	7.2		50
10	7.2	5.9		50
10	7.08	8.4		350
10	7.11	5.5		50

10	7.13	7.2		100
10	7.1	8.2	neonatal unit	50
10	7.08	6.2		1400
10	7.2	4		50
10	7.29	5.8		50
10	7.25	2.8		600
10	0	0		50
10	7.22	6.8		200
10	7.21	3.9		50
10	7.34	2.6		100
10	7.08	7.3		100
10	7.14	6.8		50
10	7.01	9.4		50
10	7.17	4.7		150
10	7.29	4.1		150
10	7.24	3.5		300
10	7.24	4.1		50
10	7.24	2.1		50
10	7	9.1		150
10	7.05	7.7		150
10	7.07	6.8		50
10	7.24	5		50
10	7.28	3.8		50
10	7.22	2.1		50
10	7.18	4.8		50
10	7.17	5.9		550
10	7.25	4.4		50
10	7.21	5.1		50
10	6.93	9		900
10	7.03	8.4		100
9	7	9.8		50
10	7.156	7		600
8	7.15	6.2		50
10	7.18	4.4		50
10	7.12	4.9		200
10	7.23	3.7		650
9	7.2	7.5		600
10	7.12	7.8		150
10	7.16	5.7		100
10	6.98	9.5		50
10	7.14	5.9		100
10	7.02	7.4		50
10	6.97	7.3		50

10	7.25	2.6	50
10	7.09	4.8	1250
10	7.31	2.6	50
10	7.26	3.1	200
10	7.14	4.4	100
