# Supplementary material for: Analysis of spontaneous labor progression of breech presentation at term
Source: PLoS One. 2022 Mar 14;17(3):e0262002. doi: 10.1371/journal.pone.0262002 (PMC8920216; doi:10.1371/journal.pone.0262002)
Supplement: Attachment — Submitted filename: Response to the reviewer final version - copie.docx [file pone.0262002.s004.docx]

PONE-D-20-40976
Analysis of spontaneous labor progression of breech presentation at term
PLOS ONE

Dear Drs.,

We would like to thank you for allowing us to revise and resubmit our manuscript to*PLOS ONE.* The reviewers’ comments are highly appreciated and have been constructive and conducive to developing an optimized manuscript. We have attached a “point-by-point” response below and made the corresponding changes to the manuscript. We believe that the changes have made our paper more suitable for publication in your esteemed journal.

Sincerely,

Dr. Benmessaoud on behalf of the coauthors

**Response**

**Reviewer #1**

**Thank you for the opportunity of reviewing this very interesting paper. The authors aimed to describe the course of labor for breech delivery with comparison according the parity and the type of breech (frank or complete). The overall message is that the course of labor is similar to the one described for vertex presentation without significant impact on the type of breech. This result is very interesting and original. It should have a clinical impact by reducing the level of intervention for stagnation in case of breech delivery and might contribute to reduce the CS rate in this situation. I only have few comments for discussion.**

Thank you for your comments and suggestions. We appreciate the time you invested in reviewing our manuscript and for providing us with an opportunity to improve it with the revised version.

**Abstract**

1. **Indicate in the abstract if the difference according parity is significant or not**

The cervical dilatation rate was slower than 1 cm/hour in the latent phase and faster than 1 cm/hour in the active phase. In the active phase, the median speed of cervical dilation was 1.67 cm/h [1.25, 2.61] (2 cm/h for multiparas and 1.54 cm/h for nulliparas). The parity difference was significant in the active phase (p<0.05).

This data has been added to the abstract.

**Main text**

2. **How do the authors interpret the surprising difference between multiparous and nulliparous women with a higher speed of dilatation in the active phase?**

We made an error when transcribing the data from Table 3 into the text. The overall dilation rate and the active phase dilation rate were faster in multiparous women. The speed of dilatation from 5 to 10 cm was 2 cm/h for multiparous women compared to 1.54 cm/h for nulliparous women, which is more logical.

3. **Does your population include women with history of CS? Which proportion? Why not including cesarean section during labor for failure of progress for a secondary analysis (at least those at full cervical dilatation) and compare the dilatation curse between those who deliver vaginally and those with CS for failure of progress?**

Regarding the data on previous cesarean sections, we did not collect this information. A history of cesarean section was not an exclusion characteristic. Cesarean sections may exist in the study’s population, but we are unable to confirm if this is the case.

We excluded patients who delivered by cesarean section during labor because we wanted to compare our curves to Zhang’s reference curves. We established our protocol according to Zhang's reference study, which excluded cesarean sections.

4. **I’d like a more important discussion about our management in labor ward. Indeed, it is widely reported that a breech delivery has to be a “fast and easy delivery”. Your results support the opposite and that finally the course of labor is not very different between a vertex or breech presentation. Discuss that your results suggest that we might consider reducing our level for intervention in case of breech delivery for stagnation of dilatation especially for this latency phase that must be respected. This might be moderate for the second stage of labor for whom a longer duration of this stage might be an alarm signal for dystocia and lead to consider CS. Provide a short and concise conclusion by the end of the conclusion**

The journal imposes a word count for a scientific article. However, we have modified the discussion and the conclusion according to your suggestions.

**Reviewer #2**

**Thank you to give me to opportunity to review this work about labor progression of breech presentation at term. However, I remain perplex because it seems difficult to conclude about labor curves from a sample of 349 breech presentation in a single center. Moreover, lacking data (or not presented in the manuscript) did not allow us to draw conclusions and extrapolate the results.**

Thank you for your comments and suggestions. We appreciate the time you invested in reviewing our manuscript and for providing us with an opportunity to improve it with the revised version.

1. **The high rate of oxytocin use (77%) limits extrapolation of the results. Author should consider to stratify analyses with and without oxytocin use.**

The population is not significant enough to allow for stratification. Stratification within the population (349 patients) will not be significant.

However, we collected times for introducing oxytocin during labor. We can add complementary data according to your recommendations. Oxytocin was introduced in the latent phase for 39 patients (14.4%), in the active phase for 150 patients (55.5%) and finally during the 2nd stage for 81 patients (30%).

The use of oxytocin is described in the protocol. Only 14.4% of patients received oxytocin in the latent phase. This result is reassuring for our practice. Recommendations to decrease oxytocin use are recent for cephalic delivery.

However, in view of the French recommendations and these first studies describing breech labor, a reflection on oxytocin use can be considered secondarily.

To consider modifications and secondary reflections, a first descriptive study, such as ours, was needed. Currently, the literature does not provide any analysis of breech labor regardless of the oxytocin rate used according to our protocol. Moreover, the low rate of neonatal and maternal complications despite a high oxytocin rate reassures us about the current use of oxytocin according to our protocol. As a reminder, oxytocin for augmentation was used in nearly half of the women included in the Consortium on Safe Labor and 74.9% in the PREMODA Study (8). It is possible that the high rate of labor augmentation reported in our study suggests more of a prophylactic than a therapeutic role.

This study’s purpose is not to provide guidelines for breech delivery but to provide a first description of breech labor and to open the discussion on vaginal breech delivery management.

We are aware that further studies are necessary.

2. **How did the author define the onset of labor from their data?**

The World Health Organization's definition of onset of labor was used for this work. The latent phase is a period characterized by painful uterine contractions and variable changes in the cervix with some degree of effacement and slow progression of dilation up to 5 cm. The active phase of labor is a period characterized by regular painful uterine contractions, a significant degree of cervical effacement and dilation up to 5 cm.

3. **The beginning of the active phase of labor is 3 cm in this paper. The author should change this threshold for 5 or 6 cm as demonstrated in Zhang’s publication.**

Like Zhang’s publication, we determined an active phase of labor at 5 cm.

In the results, we precisely state that “The first stage of labor progression was divided into a latent phase from 0 to 5 cm of dilation and an active phase from 5 to 10 cm.”

4. **I do not understand why the authors decided to exclude cesarean deliveries. Why did they not choose the censure of these data? It would be necessary to include all women reaching the second stage of labor**

Indeed, we excluded cesarean sections during labor because we established our protocol according to Zhang’s reference study, which excluded cesarean sections.

We hoped to create curves we could compare to Zhang’s reference cephalic curves. We agree that including cesarean sections during labor adds value to the statistical analysis. Caesareans could be the subject of a secondary analysis.

5. **Please describe the protocol of oxytocin use during labor in breech presentations in the center in the material section. Because this protocol is not provided, extrapolation of the data is questionable.**

The protocol is available in the appendix.

6. **Neonatal issues are also questionable. How explain the high rate of neonatal transfers: 17.5% in multiparous and 10.8% in nulliparous women. A description of transfer indications is lacking. Apgar score should be reported as percentages of newborn with 5 minutes Apgar score <7 in Table 1. Moreover, please provide information about arterial pH at birth (mean and rate <7.0, <7.10 …)**

The transfer rate is lower than national figures since the 2016 perinatal survey when the transfer rate of newborns in metropolitan France was 9.9%, which included 2.4% in child intensive care units and 4.2% in neonatology (12). In our study, 3.15% of newborns were transferred, of which 2.00% (n=7) were transferred to the neonatology unit and 1.15% (n=4) to the intensive care unit. The arterial pH was significantly lower, averaging 7.13 in nulliparous women versus 7.16 in multiparous women (p=0.013). The arterial lactate level was higher in nulliparous women: 6.54 mmol/l on average versus 5.20 mmol/l on average in multiparous women (p<0.001). Concerning the 4 newborns transferred to the intensive care unit, 3 had an arterial pH<7, and one had an arterial pH of 7.20; however, the Apgar scores were >7 at 5 min. For 7 of the newborn babies transferred to neonatology, only 1 had an Apgar score <7 at 5 min with an arterial pH of 7.05. A total of 4 of the newborn babies had a pH <7.05. Lactate levels were >10 mmol/L for 2 of them.

7. **In the results section, a description of labor management is lacking: cervical dilation at admission in labor ward unit, rate of amniotomy, cervical dilation when oxytocin was started … Without a such description of labor practices, extrapolation of these results remains questionable.**

We added these parameters to the data and to the protocol in the appendix.

8. **Table 3, last line (from 9 to 10), the significance of the comparison between complete and franck breech is probably wrong: p=0.017???**

The statistical analysis confirms these results.

9. **In the conclusion of abstract and the manuscript, please delete “when there is adequate management”. This subjective comment seems inappropriate considering the high rate of oxytocin use and the high rate of neonatal transfer.**

Management is described in the protocol. We would like to point out that the transfer rate is lower than the national transfer rate.

We collected the times for introducing oxytocin during labor. We added complementary data according to your recommendations. Oxytocin was introduced in the latent phase for 39 patients (14.4%), in the active phase for 150 patients (55.5%) and finally during the 2nd stage for 81 patients (30%).

The use of oxytocin is described in the protocol. Only 14.4% of patients received oxytocin in the latent phase. This result is reassuring for our practice. Recommendations to decrease oxytocin use are recent for cephalic delivery.

However, in view of the new recommendations and these first studies describing breech labor, a reflection on the use of oxytocin can be considered secondarily.

To consider modifications and secondary reflections, a first descriptive study, such as ours, was needed. Currently, the literature does not provide any analysis of breech labor regardless of the rate of oxytocin used according to our protocol.

Moreover, the low rate of neonatal and maternal complications despite a high oxytocin rate reassures us on the current use of oxytocin according to our protocol. Indeed, oxytocin is administered late in labor. However, a reflection on the use of oxytocin in cephalic and breech births should be pursued. However, our main objective is to counteract the high rate of cesarean section by first describing breech vaginal delivery.

This study’s purpose is not to provide guidelines for breech delivery but to provide a first description of breech labor and to open the discussion on vaginal breech delivery.

We are aware that further studies are necessary.

10. **In the introduction section, please delete in lines 93-94 “12/30/2020 2:17:00 PM” and change the police in lines 102-108**

These points have been corrected.

11. **In the materials and methods section, please provide the ethic registration number**

Under French regulations, the study was exempt from institutional ethics review because it was an observational study using anonymized data from medical records. The women were systematically informed that the obstetrical and neonatal data could be used for to evaluate medical practices and were explicitly informed that they could sign an opposition form.

**Reviewer # 3**

**The authors performed this study in order to assess cervical dilatation length during the passive and active labor of women presenting with a fetus in breech position. The primary objective was to evaluate labor progression of primiparous and multiparous women in cases of breech presentation as labor curves in such cases are not available. The secondary outcome was to determine whether the type of breech or parity can influence the speed of cervical dilatation. This retrospective study was performed in a single French center between 2010 – 2018. The inclusion criteria were: women eligible after 37 weeks of gestation with a live breech presentation if the onset of labor was spontaneous and if they completed the first and second stages with a vaginal delivery. The authors collected labor progression patterns by examining the relationship between the elapsed times from the full dilatation and cervical dilatation stages. The labor durations (min) was characterized by examining the distribution of time intervals from one cervical dilatation stage to the next and ultimately to full dilatation. The median, first quartile and third quartile of the cervical dilatation velocity were calculated.During the study period 3.3 % of the presentation were breeches. A total of 495 (76.1%) women with a breech presentation on 650 attempted a vaginal delivery. The authors illustrate the different velocities of cervical dilatation depending on parity, type of breeches and passive/active phase.** **Overall, the clinical topic is interesting as labor curves in case of breech presentation has not been previously publish. The methodology of this study is sound. Furthermore, the discussion is well constructed, and the authors address the limits of their study.**

Thank you for your comments and suggestions. We appreciate the time you invested in reviewing our manuscript and for providing us with an opportunity to improve it in the revised version.

1. **Proof-reading by native English speaker is mandatory, in order to improve its readability and correct several typos.**

The English was proofread by a certified translation agency. However, a second proofreading has been performed based on your recommendations.

2. **Introduction: Line 93-94: “12/30/2020 2:17:00 PM”. Line 101 – 108 : the police is different than from the rest of the text.**

These points have been corrected.

3. **Methods:
   Line 140: Why was the episiotomy and total volume of bleeding rate collected as it is not presented in the results?**

We selected hemorrhage and lesions of the perineum as well as episiotomy as maternal complications that occur during vaginal delivery. The results are not presented but have been added to Table 1.

4. **Table 1: Please precise if the numbers are median (IQR) or mean (SD).**

The numbers are mean (SD). This information has been added to the manuscript.

**Reviewer # 4**

**This is a unique study and is the first attempt to create labour curves for spontaneous vaginal breech delivery at term. The authors must be highly commended for this very valuable and important piece of work. As someone who is a proponent of vaginal breech this is certainly a very interesting and new approach that could be built on in the future.
However, given the single centre design, the seemingly aggressive use of syntocinon, which has not been explained, and the lack of complete outcome data, more work is needed before these curves could be used in other centers which may have more physiological approaches to childbirth.**

**Background
The authors have corrected and identified a gap in our knowledge about labour in spontaneous vaginal breech at term. They have attempted to construct normal curves for this group of women in both nulliprous and multiparous women. Another aim was to compare the curves for different types of breech presentation which is certainly quite unique. This was a retrospective single centre experience in a french maternity unit. This design gives certain advantages but also adds some limitations in terms of relevance to other units with different management protocols for breech presentation at term.**

**Abstract
The abstract is succinctly written with all the basic informations that is required for a good overview of the article.**

**Methods
The methodology is laid out very clearly and the statistical analysis is similar to Zhang’s Safe Consortium group, which has been validated.**

Thank you for your comments and suggestions. We appreciate the time you invested in reviewing our manuscript and for providing us with an opportunity to improve it in the revised version.

**I have some issues with the chosen criteria for a vaginal breech delivery attempt:**

1. **Pelvimetry is an unproven intervention with no clear advantages but I accept that in some countries it is mandatory. We lack high quality data for this intervention which can of course increase the risk of harm to the mother and the baby.**
   **The only outcome changed by pelvimetry is the rate of caesarean which unfortunately increases in the wrong way – an excuse for a caesarean for the nervous obstetrician. Probably not the case for this group but the message can be misinterpreted. I assume that multiparous women with previous normal vaginal deliveries were not subjected to this intervention? The text seems to allude that this was reserved for nulliparous only which is reassuring. This group has already performed the best test of pelvic capacity – childbirth
   So, given the lack of proof, performing a CT scan in pregnant women, thus increasing the oncogenic risk for both the mother and the baby seems somewhat illogical and without doubt breaks the ‘primum non nocere’ rule.**

**If there is a real need to do this to reassure the team then why not an MRI which carries no risk? Perhaps this was not freely available to the authors.
They also include a Magnin index which is not widely used. This is also not recommended by the CNGOF. Why was this deemed necessary?**

Pelvimetry is used in our local practice more often than the national college uses it. In our practice, it is a parametric analysis and morphological analysis of the pelvis.

Multiparous patients who have already delivered do not require pelvimetry in our center.

Pelvimetry is not systematic and is discussed according to the previous mode of delivery and the fetal weight at birth. We agree with your opinion regarding the interest in pelvic MRIs. Unfortunately, this imaging is not easily accessible in our institution. The CT scans performed are low-dose scans.

2. **The upper fetal weight estimate for an attempt was macrosomia presumably based on either a percentile of >P90 or an estimate of >4kg. It is not clear which definition has been used.
   There was no lower limit for an attempt in contrast to the PREMODA criteria. Did the authors allow inclusion of growth restricted fetuses? These were the babes that did so badly in the Term Breech Trial. This is important in order to correctly interpret the results as presented.**

Suspected fetal macrosomia was considered for a fetal estimation weight >3800 g. This information has been added to the protocol. In our hospital, we do not use a lower weight limit because the center performs breech vaginal deliveries for premature delivery.

In our population, 26 fetuses weighed <2500 g, and 7 fetuses weighed <2300 g.

3. **I am interested to know why the authors choose to present the cervical dilatation velocity as quartiles rather than percentiles: P5, P50 and P95. Not a criticism merely a question.**

We thank reviewer for raising this point. We indeed expressed cervical dilatation velocity as a median and interquartile range rather than percentiles to provide the reader with a more precise indicator of data distribution. Mean and standard deviation would not be appropriate due to the fact that velocity distribution was not always perfectly bell shaped and symmetrical. However, the interquartile range contrary to the P5 and P95 is not affected by extreme values and tells the reader more precisely how the middle 50% of the observations are spread out.

**Discussion**

4. **The study is appropriately discussed in the context of previously published work also in reference to various national organisations for vertex and non-vertex presentations at term. Lines 221-224 -the authors make an important comment on the lack of protocols for vaginal breech deliveries. A very relevant critique but not one that the authors themselves have addressed in this article, unless there is an appendix to which I do not have access. It is imperative that the specific management protocol used during the study period is made available in order to fully understand the results obtained and their relevance to other maternities.**

**o What criteria were used for the use of oxytocin augmentation ?**
**o Was this just lack of regular contractions as alluded to in the text or was thisalso based on lack of progress.**
**o What was the definition of lack of progress or stagnation/dystocia ?**
**o How frequent were the vaginal examinations – hourly, 2 hourly or 4 hourly ?**
**o Was augmentation restricted only to the active phase of labour or was it also used at less than 5 cm ?**
**o What is the policy on the use of oxytocin in the second stage of labour ? Some units in France start oxytocin systematically in the second stage if it has not already been started before. This in not normal practice elsewhere.**

The above remark is relevant. Indeed, it is necessary to specify these various elements. We answer all of the above questions in our protocol, which is available in the appendix.

We collected the times for introducing oxytocin during labor, and we added the additional data to the manuscript. Oxytocin was introduced in the latent phase for 39 patients (14.4%), in the active phase for 150 patients (55.5%), and finally during the 2nd stage for 81 patients (30%).

5. **Do the authors have more complete date for maternal and neonatal outcomes?
   No maternal data is included although episiotomy and bleeding is mentioned in the methodology section, so I assume that this data was collected.
    More neonatal data is required. This is important as the very impressive vaginal delivery success rate of 81% could have come at a significant cost to both the mother of the baby**

Indeed, we collected data on maternal complications concerning perineal lesions and postpartum hemorrhage.

These data were removed to correspond with the review criteria that limited the amount of information. We added this information to the manuscript.

To expand neonatal data, we added data concerning the average pH and lactate level at birth according to parity.

The transfer rate is lower than national figures since the 2016 perinatal survey when the transfer rate of newborns in metropolitan France was 9.9%, which included 2.4% in child intensive care units and 4.2% in neonatology (12). In our study, 3.15% of newborns were transferred of which 2.00% (n=7) were transferred to neonatology and 1.15% (n=4) to intensive care.We compared the average using parity.

The arterial pH was significantly lower, with an average of 7.13 in nulliparous women versus 7.16 in multiparous women (p=0.013). The arterial lactate level was higher in nulliparous women: 6.54 mmol/L on average versus 5.20 mmol/L on average in multiparous women (p<0.001).

Concerning the 4 neonates transferred to the intensive care unit, 3 had an arterial pH <7, and one had an arterial pH of 7.20, but its Apgar score was <7 at 5 min.

For 7 of the neonates transferred to neonatology, only 1 had an Apgar score <7 at 5 min with an arterial pH of 7.05. A total of 4 of the neonates had a pH <7.05. Lactate levels were >10 mmol/L for 2 of them.
